# Supplementary material for: Developmental phenomics suggests that H3K4 monomethylation confers multi-level phenotypic robustness
Source: Cell Rep. 2022 Dec 13;41(11):111832. doi: 10.1016/j.celrep.2022.111832 (PMC9764455; doi:10.1016/j.celrep.2022.111832)
Supplement: Document S1. Figures S1–S5 and Table S1 [file mmc1.pdf]

Cell Reports, Volume *41*

Supplemental information

Developmental phenomics suggests that H3K4 monomethylation confers multi-level phenotypic robustness

Lautaro Gandara, Albert Tsai, Måns Ekelöf, Rafael Galupa, Ella Preger-Ben Noon, Theodore Alexandrov, and Justin Crocker

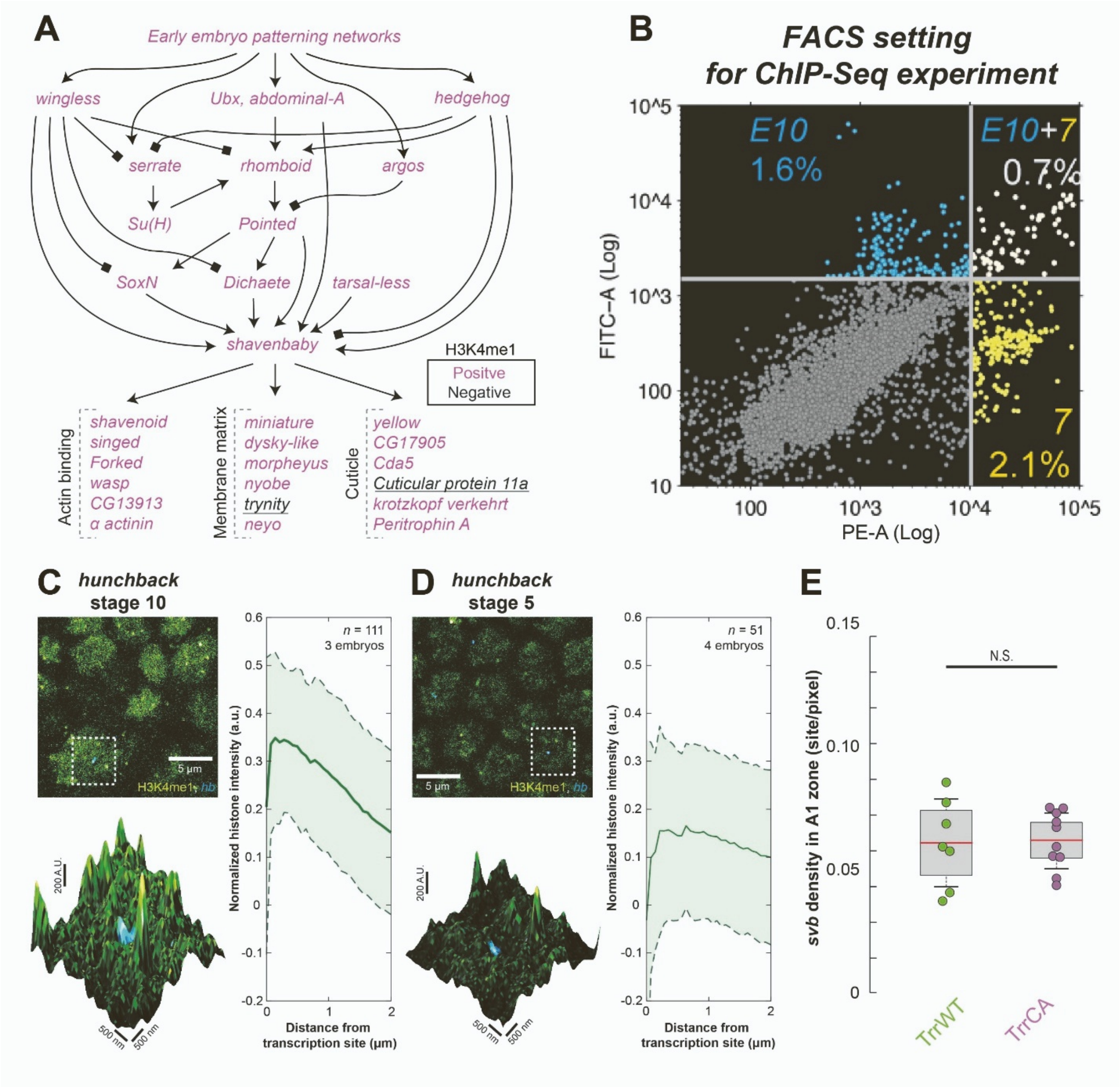

Supplementary Figure 1. H3K4me1 and *svb* transcriptional robustness. Related to Figure 1.

(A) H3K4me1 deposition near developmental transcription factors in the *shavenbaby* regulatory network (the presence of H3K4me1 peaks within 10 kb of the start site for each of the genes in the network is indicated as "positive" and highlighted in magenta).

(B) The setting used to sort cells in FACS for the ChIP-Seq experiment shown in Figure 1B.

(C & D) High resolution confocal imaging experiments in w^{1118} embryos showing the distribution of H3K4me1 in *hb* transcription sites (C for stage 10 and D for stage 5 embryos). The lower panels show a zoomed-in view from the dotted boxes with the height indicating the intensity of the H3K4me1 signal. The normalized average H3K4me1 intensity over multiple transcription sites (C: n=111; D: n=51) is shown on the plots at the right. The shaded region is the variance. C & D were adapted from (Tsai and Crocker, 2022).

(E) Density of *svb* transcription sites in the first abdominal (A1) segment of stage 15 embryos at 25 °C within the ventral band where *svb* is normally expressed (compare with Figure 1H). The number of embryos are: 10 for TrrWT and 7 for TrrCA. The boxed region is one s.d. and the tails are two s.d. (95 %). N.S.: not significant using two-tailed Student's *t*-test

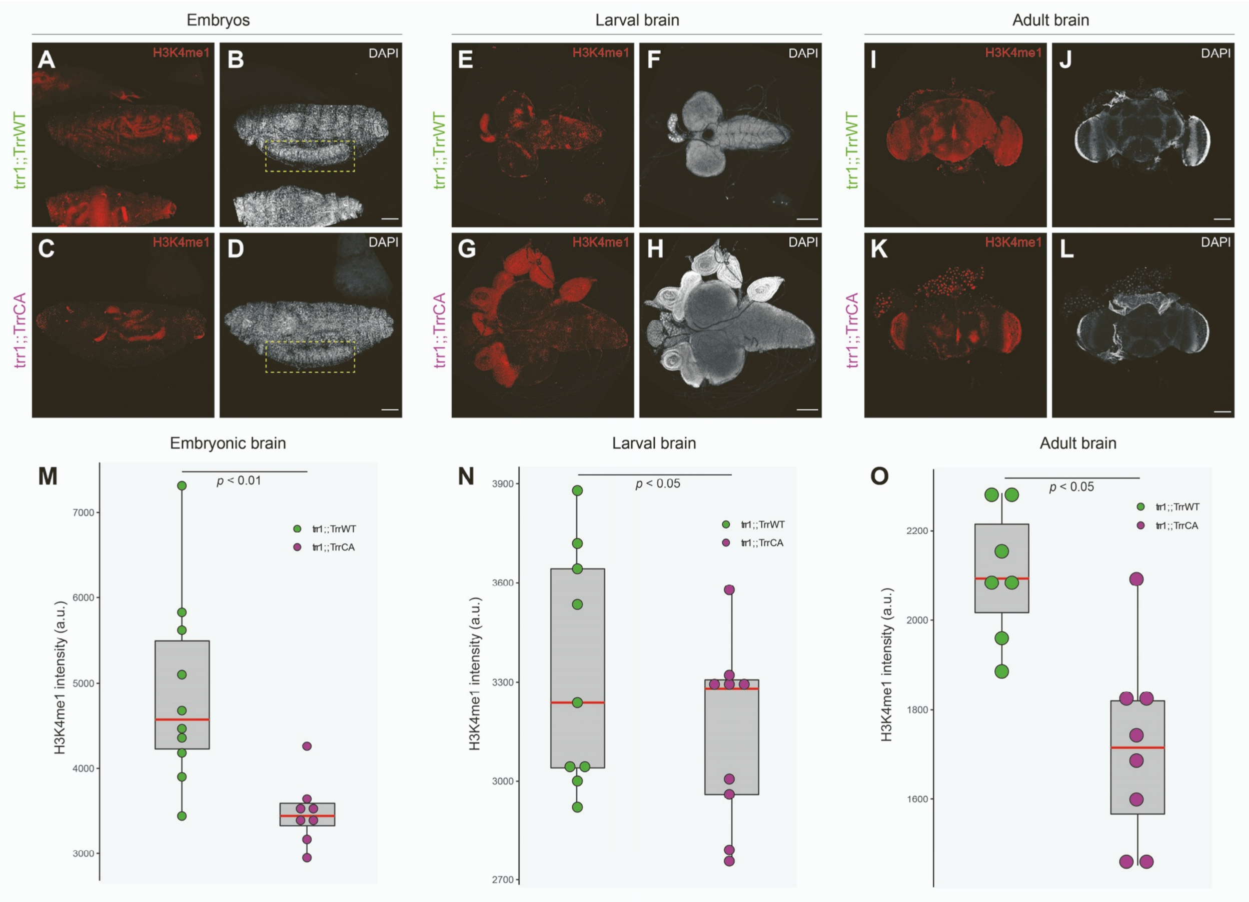

Supplementary Figure 2. H3K4me1 hypomethylation in the central nervous system produced by the TrrCA allele persists throughout the entire life cycle. Related to Figure 1.

(A – L): Immunostaining showing the H3K4me1 signal (A, C, E, G, I & K), or (B, D, F, H, J & L) DAPI staining, in embryonic (A – D, ventral nerve cord is highlighted with yellow dashed boxes), larval (E – H) and adult (I – L) brains. Scale bars: 50 µm in B and D, and 100 µm in F, H, J and L.

(M – O): Intensity of the H3K4me1 signal in embryonic (n=10 for TrrWT and n=8 for TrrCA), larval (n=9 for TrrWT and n=9 for TrrCA) or adult (n=7 for TrrWT and n=8 for TrrCA) brains of *trr1*;;TrrWT or *trr1*;;TrrCA.

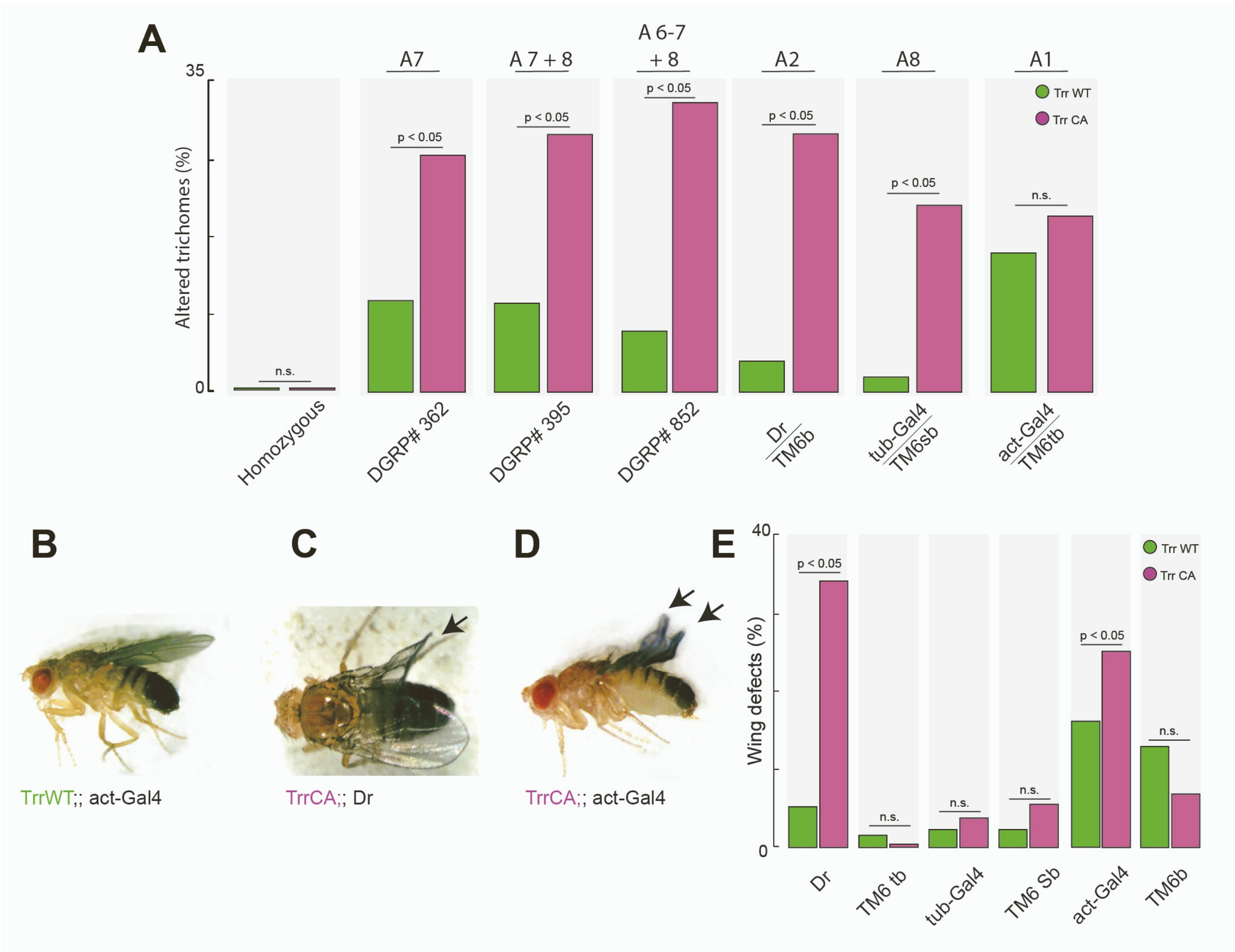

Supplementary Figure 3: Aberrant morphologies in hypomethylated flies with different genetic backgrounds. Related to figure 2.

(A) Frequencies of altered trichome patterns detected in the offspring of the crosses of the *trr*^{1} mutant lines with the DGRP lines or balancer stocks. Only the genotype-specific alterations shown in Figure 2E-L were considered for the quantification. n=52 for TrrWT (homozygous), n=35 for TrrCA (homozygous), n=54 for TrrWT x DGRP#362, n=27 for TrrWT x DGRP#395, n=56 for TrrWT x DGRP#852, n=59 for TrrCA x DGRP#362, n=11 for TrrCA x DGRP#395, n=48 for TrrCA x DGRP#852, n=113 for TrrWT x TM6/act-G4, n=156 for TrrWT x TM6/tub-G4, n=45 for TrrWT x TM6/Dr, n=62 for TrrCA x TM6/act-G4, n=51 for TrrCA x TM6/tub-G4, n=57 for TrrCA x TM6/Dr.

(B-D) Pictures of adult trr^1 mutant flies with normal wings from TrrWT, or aberrant wing morphologies (arrows) from TrrCA.

(E) The fraction of flies with deformed wings observed in each of the previously mentioned crosses. n=116 for TrrWT;;act-gal4, n=112 for TrrWT;;TM6 tb, n=152 for TrrWT;;tub-Gal4, n=153 for TrrWT;;TM6 Sb, n=117 for TrrWT;;Dr, n=40 for TrrWT;;TM6b, n=34 for TrrCA;;act-gal4, n=72 for TrrCA;;TM6 tb, n=48 for TrrCA;;tub-Gal4, n=93 for TrrCA;;TM6 Sb, n=19 for TrrCA;;Dr, n=26 for TrrCA;;TM6b

All *p*-values in the figure are from Chi-Square goodness of fit tests. n.s.: not significant.

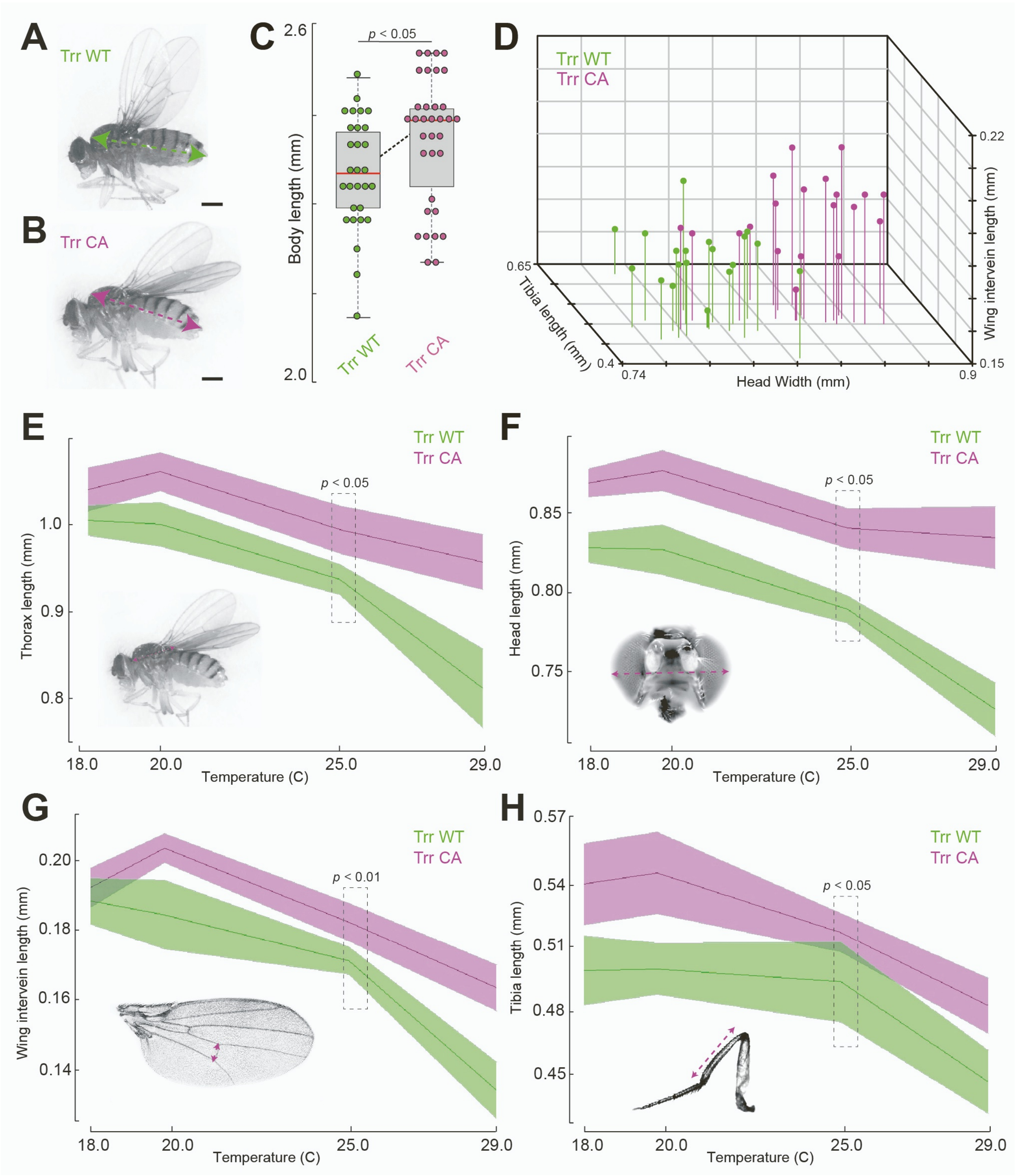

Supplementary Figure 4: H3K4me1 controls body size in adults. Related to Figure 3.

(A & B) Pictures of 72 h old (after emergence) adult flies from TrrWT (A) or TrrCA (B) (scale bar = 0.5 mm). The dashed lines show the measured body length.

(C) The body length of TrrCA flies versus TrrWT (n=29 for TrrWT and n=34 for TrrCA). Center line, mean; upper and lower limits, s.d.; whiskers, 95% CIs.

(D) 3D plot showing the values from panels d to f but linked to single individuals from TrrWT or TrrCA (n=21).

(E - H) Length of the thorax (E), the head width (F), the posterior cross-vein (G), and the fore tibia (H) in both TrrWT and TrrCA adult flies developed at different temperatures(n=20). Center line, mean; upper and lower limits, s.d.

All *p*-values are from two-tailed Student's *t*-test comparing the two trr^1 lines.

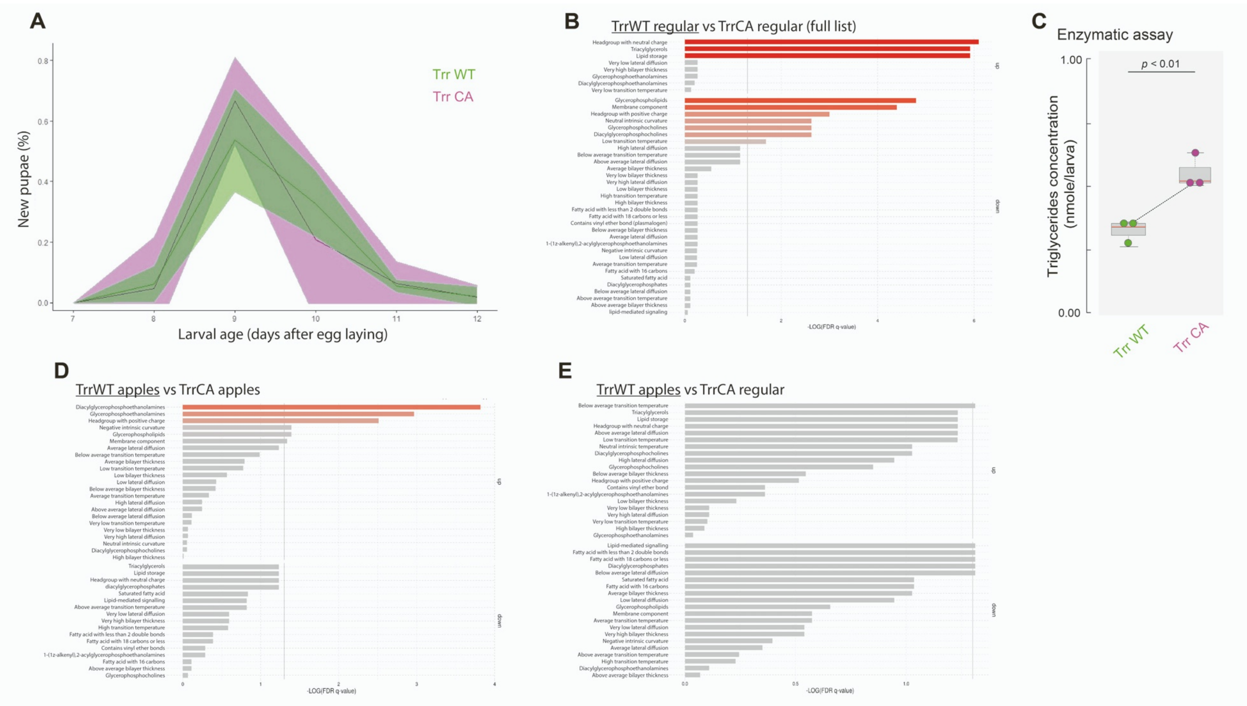

Supplementary Figure 5: Metabolomics of the *trr*^{1} lines. Related to Figure 3.

(A) Pupariation time in both *trr*^{1} mutant lines. The middle line shows the mean value for that time point, whilst the ribbon describes the CI (α=0.05); N (number of tested populations per condition) = 3.

(B) Enrichment analysis comparing both *trr*^{1} mutant lines, based on the abundance values for 77 metabolites consistently detected in all tested conditions (full list)

(C) Enzymatic determination of triglyceride levels in TrrWT and TrrCA larvae with the same time of development (120 h). N=3. Centre line, mean; upper and lower limits, s.d.; whiskers, 2.s.d. 95% CIs. Two-tailed Student's *t*-test comparing the two *trr*^{1} lines.

(D & E) Enrichment analysis from pairwise comparisons between *trr*^{1} mutant lines on apple-based food (D) and between the two populations with strong overlap in the PCA, TrrWT on apples and TrrCA on standard food (E).

gRNA1	GTCAAAGTTACAAGAAAACATGG
gRNA2	GCTATATGCAGATATGCTCGTGG
Template	TCAGAGCTAAGCAACCTGCTCCGTGTGGGCAACATGACGTTCCTGAACGTGGGTCAGTTACTGCCGCATCAGC<br>TGGAGGCCTTCCACACACCCCACTACATCTACCCCATTGGCTACAAGGTGGTAAGTACAAGTACCAGCCGGTC<br>GGCCGCCATGTTTATGAACAGCAATCTTAAATCCCGTTCCTCTTCCTCCCCCATAGAGTCGCTACTACTGGTGCG<br>TGCGACGACCCAATCGTAGGTGTCGATACATTTGCAGTATCGCAGAGGCCGGATGCAAGCCCGAGTTTCGCAT<br>CCAGGTGCAGGACGCCGGCGACAAGGAGCCGGAGCGCGAGTTTCGGGGCAGTTCGCCCTCGGCGGTCTGGC<br>AGCAGATTCTGCAGCCAATCACGCGACTGCGTAAGGTGCACAAGTGGTTGCAGCTCTTTCCCCAGCACATCAG<br>TGGTGAGGACCTATTTGGCCTAACGGAACCGGCCATTGTGCGAATCCTGGAAAGCCTCCCGGGCATTGAGAC<br>GCTCACTGACTACCGCTTCAAGTACGGTCGCAACCCGCTTCTTGAGTTCCCGCTAGCCATCAATCCGTCTGGAG<br>CGGCGCGTACGGAACCCAAGCAACGACAGCTGCTCGTCTGGAGGAAGCCGCATACGCAACGCACGGCCGGCA<br>GCTGCAGCACGCAGCGGATGGCCAACTCGGCTGCGATTGCCGGAGAAGTGGCCTGCCCGTACAGCAAGCAGT<br>TCGTGCATTCGAAGAGCTCCCAGTACAAAAAGATGAAGCAGGAGTGGCGCAACAACGTCTACTTGGCGAGGT<br>GAGACATTAGGTCCTAGTCAATAACCACTGATTACTTAAGCTCTCATTTTCTAGATCTAAGATCCAGGGTCTTG<br>GCTTGTATGCAGCTCGGGACATTGAAAAGCACACCATGATAATCGAGTACATTGGCGAGGTTATCCGCACCGA<br>GGTCTCCGAGATACGCGAGAAGCAGTACGAGTCCAAGGTGGGTTTCGCGACCATGCTTCCTTGTAACTTTGAC<br>TAATCTCTGATTCCCCAGAACCGTGGCATCTACATGTTCCGGCTGGATGAAGACCGCGTGGTGGACGCCACTCT<br>AAGCGGCGGGTTGGCGCGCTACATCAACCATTCGTGCAATCCCAACTGTGTTACCGAGATAGTGGAGGTCGAT<br>CGCGACGTGCGGATCATAATATTCGCCAAGCGGAAGATCTATCGCGGCGAGGAGGTGGGTCCCGCAAATAGA<br>AGTGTTTCGCCTGTACCTTAACTCACAATATCTCTTAAATTCTGTGCTTTAGCTCTCGTACGACTACAAGTTCGAC<br>ATTGAGGACGAGTCGCACAAGATACCG**GCC**GCCTGTGGAGCGCCCAACTGTCGCAAGTGGATGAACTAGAGT<br>CGGGAGGAGCCTCTAGCGGAGGACCAAAGGAAGCGCAAGAAGCGGCGGCGAGCACGTCGCTCCCAGTGAAC<br>AGGAACAGGAGTAGAGACAGGAACAGGAACAGGACTTGTATCTGATCAAGTAGCTAGATTCGCTAAGCATTA<br>TAGTTCGTGTACATTTATATATATATATATATATATATATATATATATTTATATATATATAGTTTATATGCTATATGC<br>AGATACGCCCGTGGAGAGCATATGACCTTGTAAGTTTTGTATATGTGCCCACTGTAGCCAATTCGTATCCTTTA<br>TATCGCTCTTAAGTGCAAAAGTGGAGAGTGTCTTCATAGTTTAAATTGAATAATTGCTATCGTTTCTACATAGA<br>AAGAATATAAATGTTACTGCTAGGACACGACCACCCCTGTAATGTATTTCGGACCCCCTCTCCTCACACAGTCG<br>CTGGAGCACCTAATTTTATTTGTTTTAGCACCATCTCCACATTCATCACTACCACCATTGCAATTATCGCGAGGA<br>AACTGACCGCCCCGATGGCCAACCCAAAACTTGACTTTATGCGTGTGTGCCTTCCCCTCTGTTTACCATTATTAT<br>TATTATCATAATTGTAGCGGTTATCTTTCAATATTCGGCCACTTTACATGGGGAGCGATCCTTCTTTCGCTGCGT<br>GTATCGTGCGCAACCTTCTTTGAATATATACACTGATAAGGATATGAACACCTACTTCTATTTTATTTTATTTAAA<br>TAGTTTGCGATTACTTTTAAGTACATTAACTAACTCCGAACAACGTGTATTTTTCATCCCCTTAAATAGTAGCTA<br>AGTAAATTTTATGCCACCATTTTACGCACGTGATCTTTTATTCCAACTTATTGCAAACGGCAAACTGGAAATTAC<br>GAAATGGGGACGACAGATATTGCACCTAAGATCTTCGCTTGGCCAGCGCCAGAAGCCTCTGGAGCTGGACTG<br>CGGCAGCCAGGGTGAGTATGTCGAAGTTCTTCAGGTACCAGGAGATGTTGTACATGATGTGGATGCGCCAGT<br>TCTTGTCGAGGAACGCTTTGTAGAAGTGGCTCATCTGGTCCGCGGACACTTCGCTGGTTCCGCTTTCCTTGTGC<br>AGGCGGATGAAGTCTTCCTTTTCCTGTAAACGAACCAATTCGAAAGAGACGACATTAGCGGACTAGAAAGAGA<br>TGGCACAGTGGTCGTGGTTGTACCTCGTAGAAGCGCTTATTGTGTTTTGT

Supplementary table 1. Sequences employed to build a new TrrCA allele using CRISPR/Cas9. Related to Figure 4.

Sequences for the two guides and the template DNA that were required to edit the native *trr* locus using CRISPR/Cas9. The nucleotides in red are the ones that were altered to turn the Trr protein catalytically deficient by replacing the cysteine at position 2398 by an alanine. Guide-related sequences in the template are shown in green.
