# Supplementary material for: Developmental phenomics suggests that H3K4 monomethylation confers multi-level phenotypic robustness
Source: Cell Rep. 2022 Dec 13;41(11):111832. doi: 10.1016/j.celrep.2022.111832 (PMC9764455; doi:10.1016/j.celrep.2022.111832)
Supplement: Document S2. Article plus supplemental information [file mmc2.pdf]

Cell Reports

Report

Developmental phenomics suggests that H3K4 monomethylation confers multi-level phenotypic robustness

Graphical abstract

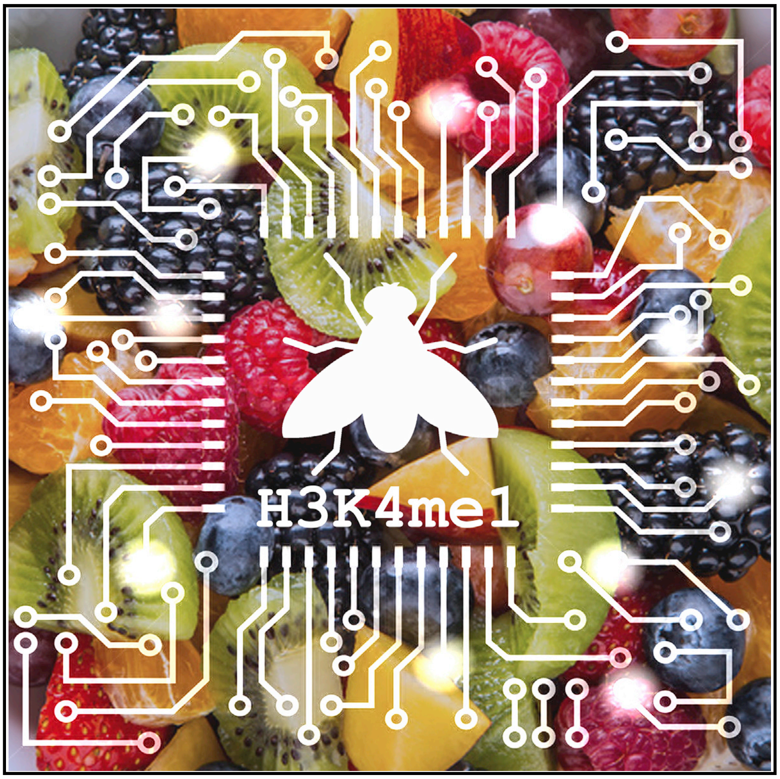

Authors

Lautaro Gandara, Albert Tsai, Måns Ekelöf, Rafael Galupa, Ella Preger-Ben Noon, Theodore Alexandrov, Justin Crocker

Correspondence

albert.tsai@embl.de (A.T.),
justin.crocker@embl.de (J.C.)

In brief

Gandara et al. leverage phenomics to show that H3K4me1 has a pervasive function in *Drosophila* biology. On a mechanistic level, this histone mark supports the establishment of nuclear microenvironments and improves transcriptional robustness. Hypomethylation results in a loss of phenotypic robustness affecting metabolism, behavior, and offspring production.

Highlights

- H3K4me1 supports the establishment of transcriptional nuclear microenvironments
- Its absence leads to altered gene expression and loss of phenotypic robustness
- ΔH3K4me1 fruit flies show diverse altered phenotypes under sub-optimal conditions
- The affected phenotypes include metabolism, behavior, and offspring production

Gandara et al., 2022, Cell Reports *41*, 111832
December 13, 2022 © 2022 The Authors.
https://doi.org/10.1016/j.celrep.2022.111832

CellPress

Cell Reports

CellPress
OPEN ACCESS

Report

Developmental phenomics suggests that H3K4 monomethylation confers multi-level phenotypic robustness

Lautaro Gandara,[1,6] Albert Tsai,[1,6,*] Måns Ekelöf,[2] Rafael Galupa,[1] Ella Preger-Ben Noon,[3] Theodore Alexandrov,[2,4,5] and Justin Crocker[1,7,*]
[1]Developmental Biology Unit, European Molecular Biology Laboratory, Heidelberg, Germany
[2]Structural and Computational Biology Unit, European Molecular Biology Laboratory, Heidelberg, Germany
[3]Department of Genetics and Developmental Biology, The Rappaport Faculty of Medicine and Research Institute, Technion-Israel Institute of Technology, Haifa, Israel
[4]Molecular Medicine Partnership Unit, European Molecular Biology Laboratory, Heidelberg, Germany
[5]BioInnovation Institute, Copenhagen, Denmark
[6]These authors contributed equally
[7]Lead contact
*Correspondence: albert.tsai@embl.de (A.T.), justin.crocker@embl.de (J.C.)
https://doi.org/10.1016/j.celrep.2022.111832

SUMMARY

How histone modifications affect animal development remains difficult to ascertain. Despite the prevalence of histone 3 lysine 4 monomethylation (H3K4me1) on enhancers, hypomethylation appears to have minor effects on phenotype and viability. Here, we genetically reduce H3K4me1 deposition in *Drosophila melanogaster* and find that hypomethylation reduces transcription factor enrichment in nuclear microenvironments, disrupts gene expression, and reduces phenotypic robustness. Using a developmental phenomics approach, we find changes in morphology, metabolism, behavior, and offspring production. However, many phenotypic changes are only detected when hypomethylated flies develop outside of standard laboratory environments or with specific genetic backgrounds. Therefore, quantitative phenomics measurements can unravel how pleiotropic modulators of gene expression affect developmental robustness under conditions resembling the natural environments of a species.

INTRODUCTION

Gene regulation across animal development occurs through networks of interacting transcription factors and is modified by the cellular environment, biochemical pathways, metabolic state, and additional elements summarized in Waddington's metaphor of epigenetic landscapes (Davidson and Levine, 2008). These networks are the products of evolution, changing in response to external conditions (Scheiner, 2002). To gain traction into this complexity, a classical approach has been to use lab-bred model organisms, standardize the experiments under controlled conditions, and measure pre-determined variables that are expected to change. Such a reductionist approach has successfully dissected essential components and their interactions across development.

However, such an approach may not fully reveal how systems function in their native environments (Bergelson et al., 2021). Recent advances have enabled a high-throughput, complementary approach: the unbiased and unconstrained exploration of multiple, complex phenotypes and environmental conditions. Spatially resolved mass spectrometry and automated video tracking enable quantitative and cost-effective explorations of metabolism and behavior (Houle et al., 2010). Furthermore, acquisition of high-dimensional phenotypic data, or "phenomics" (Houle et al., 2010), could study nuanced modulators of gene expression or robustness-conferring elements (i.e., elements that reduce the effect of mutation, environmental stimuli, and/or noise on gene expression [Masel and Siegal, 2009]), revealing their impacts on the entire organism and populations.

The monomethylation of histone H3 on lysine 4 (H3K4me1) has disputed roles (Rada-Iglesias, 2018) in gene regulation—while it is associated with enhancer elements (Barski et al., 2007; Bonn et al., 2012; Heintzman et al., 2009) across many species (Bogdanović et al., 2012; Bonn et al., 2012; Nègre et al., 2011), its loss appears to have minor effects. In mouse embryonic stem cells, the loss of H3K4me1 in Mll3/4 catalytically deficient cells had minimal effects on transcription (Dorighi et al., 2017; Lee et al., 2013) and self-renewal (Dorighi et al., 2017). While H3K4me1 may have relevance in mouse development (Bleckwehl et al., 2021; Xie et al., 2020), hypomethylation through disrupting the catalytic activity of Trithorax-related (Trr), the main methyltransferase behind H3K4me1 in *Drosophila melanogaster* (Herz et al., 2012), did not affect development or viability. Subtle morphological defects could be observed only when raising the

© 2022 The Authors.
This is an open access article under the CC BY license (http://creativecommons.org/licenses/by/4.0/).

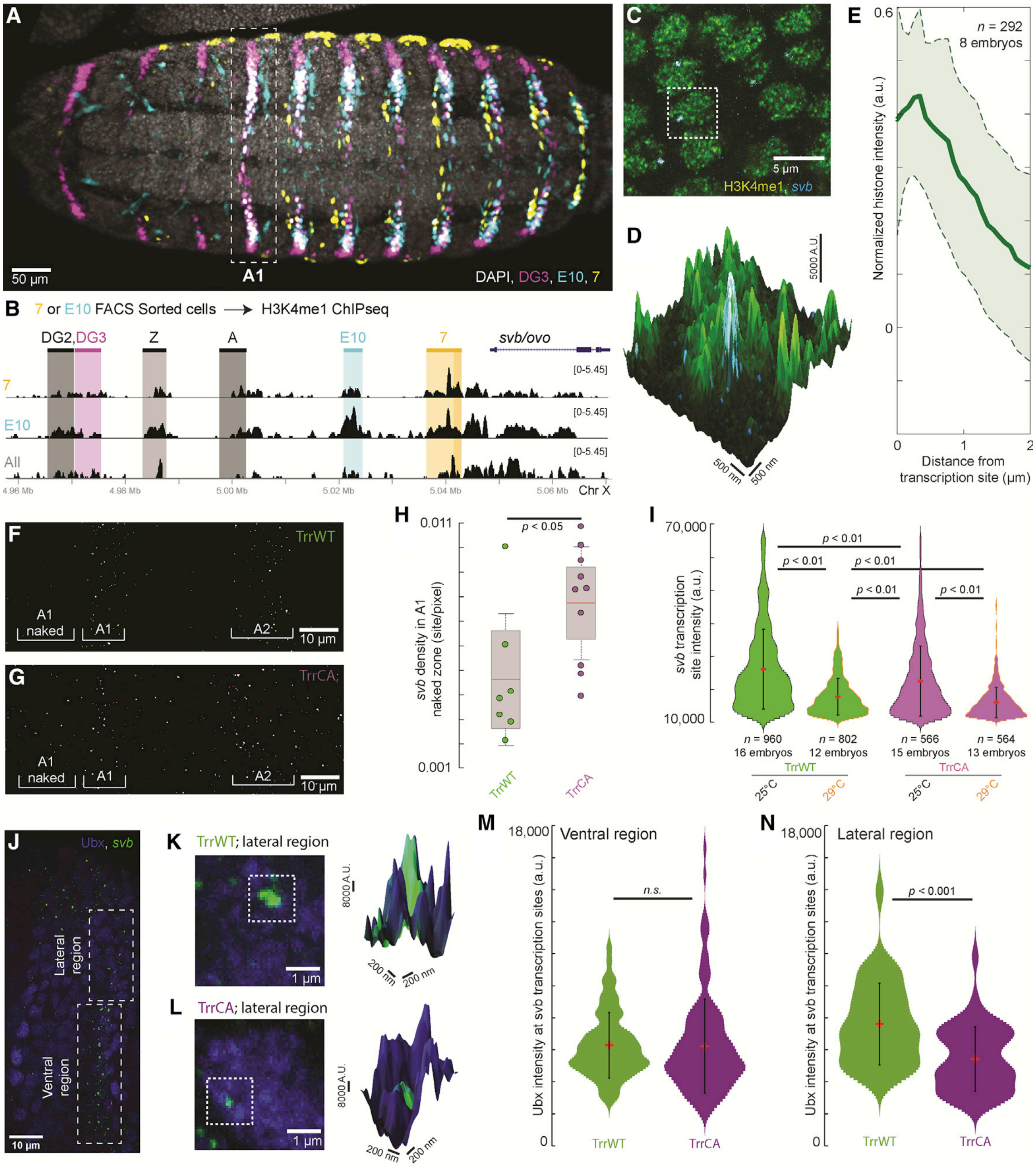

Figure 1. H3K4me1 at the *svb* locus supports transcriptional robustness and microenvironment integrity

(A) Ventral view of a stage 15 *Drosophila melanogaster* embryo from the fly line used for the ChIP-seq experiment stained for the products of the reporter genes driven by the *svb* enhancers *DG3*, *E10*, and *7*. The white dotted box is the first abdominal (A1) segment.

(B) ChIP-seq using cells from stage 15 *Drosophila melanogaster* embryos sorted by reporter gene activity. The panel shows H3K4me1 enrichment at the *svb/ovo* locus, in cells with an active *7* enhancer (orange), an active *E10* enhancer (cyan), or from the entire embryo ("All," gray). The darker shade in the *7* enhancer highlights the "H" region, which encompasses most of its reported regulatory activity, where the enrichment of H3K4me1 is most conspicuous.

(C) Confocal imaging experiments in stage 15 w^{1118} embryos show that *svb* transcription sites are in regions enriched for H3K4me1. The dashed box highlights a single nucleus of the embryonic epidermis.

(D) Zoomed-in view of a single nucleus (dotted box in C) with the height indicating the intensity of the H3K4me1 signal. *svb* RNA is in cyan.

(E) Normalized average H3K4me1 intensity over 292 transcription sites in 8 embryos. The shaded region is the variance.

(F and G) *svb* transcription sites at 25°C on the ventral side of the first two abdominal (A1 and A2) segments of stage 15 embryos in both trr^1 lines.

(H) Transcription site density in front of the A1 ventral band ("A1 naked"). Number of embryos: 7 (TrrWT) and 10 (TrrCA). The center line is the mean. The boxed region is one SD, and the tails are two SDs (95%).

(I) Intensity of *svb* transcription sites at different temperatures. The red dot is the mean, and the bar is two SDs.

(legend continued on next page)

flies at elevated temperatures (Rickels et al., 2017). Of note, the impairment of Trr catalytic activity had only mild effects on H3K4me2 and H3K4me3, as other SET-domain-containing proteins in *Drosophila* (dSet1 and Trx) also maintain these histone marks (Mohan et al., 2011). The lack of clearly defective phenotypes has therefore generated the hypothesis that H3K4me1 fine-tunes enhancers for a more nuanced response to environmental or genetic stresses (Gibert et al., 2016; Rickels et al., 2017). However, this subtle effect contrasts with the presence of H3K4me1 throughout the *Drosophila* genome (Bonn et al., 2012; Rada-Iglesias, 2018).

To measure the effects of H3K4me1 on phenotypes, we designed a developmental phenomics workflow (Houle et al., 2010) and applied it to a *D. melanogaster* line with deficient Trr activity, challenging it with various genetic and environmental conditions. Starting from a single regulatory network, we demonstrated that H3K4me1 may confer transcriptional robustness by preserving transcriptional microenvironments in the nucleus. Then, we assessed the systematic impact of H3K4me1 hypomethylation in larvae using multiple phenotypic assays. Consistent with the ubiquitous presence of H3K4me1 across the genome (Barski et al., 2007; Bonn et al., 2012; Heintzman et al., 2009), hypomethylation altered morphology, metabolism, behavior, and production of adult offspring in response to genetic and environmental challenges. In sum, global H3K4me1 hypomethylation reduced developmental robustness and revealed phenotypic variations depending on environmental and genetic contexts, potentially altering the evolutionary response of *Drosophila* populations to specific environments.

RESULTS

Transcriptionally active *shavenbaby* (*svb*) loci have enriched levels of H3K4me1

Previous works showed that global patterns of gene expression were unaffected by H3K4me1 hypomethylation (Dorighi et al., 2017; Rickels et al., 2017). However, H3K4me1 exhibited distinct trends between its global nuclear distribution and enrichment around individual genes compared with other histone modifications during embryo development (Tsai and Crocker, 2022), suggesting that it serves specific regulatory functions. As an increase in H3K4me1 was associated with active enhancers (Bonn et al., 2012) and with activity across the *svb* network controlling trichome development in ectodermal cells (Figure S1A; see correlation between H3K4me1 deposition and the regulation of the svb network in the STAR Methods), we investigated if transcriptionally active *svb* loci show enrichment for H3K4me1. To capture cells with active ventral *svb* enhancers, we fluorescently activated cell sorted (FACS) nuclei from stage 15 *Drosophila melanogaster* embryos using the expression of reporter genes driven by *svb* enhancers: "*E10*" driving GFP and "*7*" driving *dsRed* (Figure 1A). The sorted nuclei were then processed through chromatin immunoprecipitation sequencing (ChIP-seq) targeting H3K4me1 (Figures 1B and S1B). As expected, H3K4me1 marked the known embryonic enhancers of *svb* in nuclei from the entire embryo ("All," Figure 1B). Cells where the reporter for a specific *svb* enhancer is active ("*7*" or "*E10*," Figure 1B) showed increased monomethylation over the corresponding enhancer and across the *svb cis*-regulatory region.

We performed high-resolution confocal imaging along the ectoderm in the first abdominal (A1) segment (white box in Figure 1A) of stage 15 embryos (w^{1118}) to see if H3K4me1 is locally enriched at active *svb* loci. We located cells that are expressing *svb* using fluorescence *in situ* hybridization (FISH) with RNA probes targeting the *svb* mRNA (Tsai et al., 2019) and stained for H3K4me1 using immunofluorescence (IF). As previously described (Tsai et al., 2017), *svb* transcription sites appear as discrete spots with high signal intensity, indicating a large number of mRNA molecules (Figures 1C and 1D; sample preparation and staining for confocal imaging in the STAR Methods). The average radial intensity distribution of H3K4me1 as a function of distance from the transcription site showed that *svb* transcription sites sit on a local maximum (Figure 1E), reminiscent of Ubx concentrations around *svb* (Tsai et al., 2017). This local enrichment of H3K4me1 at *svb* sites was stronger than previously observed H3K4me1 enrichment at *hb* transcription sites (Tsai and Crocker, 2022) (Figures S1C and S1D, adapted from Tsai and Crocker, 2022). Thus, ChIP-seq and imaging both suggest that H3K4me1 is enriched at transcriptionally active *svb* enhancers.

Hypomonomethylation of H3K4 lowered the transcriptional output of *svb*

To identify the effects of losing H3K4me1 on *svb* expression, we used a previously characterized fly line with the trr^1 null allele complemented with a construct bearing a cysteine-to-alanine (C2398A) mutation ((trr^1;;*trr*(C2398A)), "TrrCA"). This reduced H3K4me1 deposition throughout the life cycle of the animal (Figure S2) but rescued trr^1-induced lethality (Rickels et al., 2017). This TrrCA line produced fertile adults with a normal life span, no gross morphological abnormalities, and normal gene expression in adult brains and larval wing imaginal discs compared with control lines (Rickels et al., 2017). Interestingly, these flies showed subtle changes in their wing venation pattern when developed at 29°C—a non-optimal condition for *Drosophila* development (Rickels et al., 2017). We used the trr^1 null line rescued with the wild-type Trr ((trr^1;;*trr*(WT)), "TrrWT") as our control to rule out effects from the trr^1 line.

(J) Confocal microscopy image of active *svb* transcription sites and Ubx distribution in the A1 segment of a stage 15 TrrWT embryo.
(K and L) Confocal imaging experiments in stage 15 embryos show that H3K4me1 hypomethylation impairs Ubx enrichment at *svb* transcription sites. Right panels: zoomed-in view of the dotted boxes with the height indicating the intensity of the Ubx signal.
(M and N) Intensity of the Ubx signal in *svb* transcription sites measured in the ventral (M) or in the lateral region (N). The red dot is the mean, and the bar is two SDs. Number of embryos: TrrWT = 5 embryos, TrrCA = 8 embryos. Number of analyzed transcription sites in the ventral region (M): TrrWT n = 69 and TrrCA = 139 and in the lateral region (N): TrrWT n = 38 and TrrCA n = 45.
All p values in the figure are from two-tailed Student's t tests. n.s., not significant.
Related to Figures S1 and S2.

To observe how hypomethylation changes *svb* regulation, we quantified *svb* transcription sites in the A1 segment of stage 15 embryos using FISH. Even at 25°C, the TrrCA line had numerous transcription sites outside of the ventral stripes, while there were fewer in the TrrWT line (Figures 1F and 1G): the region in front of the A1 stripe had an average of 0.0077 sites per pixel in TrrCA versus 0.0046 in TrrWT (Figure 1H). While the density of transcription sites within the A1 ventral stripe was similar between TrrCA and TrrWT (Figure S1E), the intensity of *svb* transcription sites in TrrCA was lower than TrrWT at 25°C (Figure 1I). At 29°C, *svb* transcription site intensity decreased for both lines; however, it was again lower in the TrrCA line (Figure 1I).

These *svb* transcription sites normally reside inside transcriptional microenvironments, which are locally enriched for transcription factors (TFs) required for *svb* expression (Tsai et al., 2017). Thus, we analyzed if hypomethylation affects the enrichment of the TF Ubx, the Hox factor driving ventral *svb* expression in the A1 segment (Figures 1J–1N). Ubx intensity at *svb* transcription sites was similar between TrrWT and TrrCA in the ventral region (Figures 1J and 1M). However, in the lateral region, where *svb* transcription is driven by a single enhancer, *DG3*, and trichome development is less robust (Tsai et al., 2019), Ubx intensity was reduced in TrrCA (Figures 1K, 1L, and 1N). In sum, H3K4me1 hypomethylation reduced both the accuracy and levels of *svb* expression and, in the absence of enhancer redundancy, impaired local *svb* transcriptional microenvironments.

Reduced H3K4me1 impaired the robustness of trichome phenotype at increased temperatures

Based on the effect of H3K4me1 hypomethylation on *svb* transcription, we analyzed trichome development under the control of the *svb* network. At 25°C, TrrWT larvae had no trichomes outside of the ventral band (Figure 2A). In contrast, TrrCA larvae developed extra trichomes outside of the ventral band (Figure 2B, arrows). While TrrWT larvae had similar numbers of A1 ventral trichomes at 25°C, 29°C, and 32°C, the number of trichomes progressively dropped in the TrrCA line as the temperature increased (Figure 2C). Similar trends were observed at the lateral edge, where *svb* regulation depends on a partially different set of regulatory elements (Frankel et al., 2010); however, here, the TrrCA line had fewer trichomes than TrrWT at all temperatures tested (Figure 2D). These results indicate that H3K4me1 hypomethylation reduces the robustness of trichome patterning at increased temperatures, which is consistent with observations that H3K4 hypomethylation led to environment-dependent phenotype alterations (Rickels et al., 2017).

H3K4me1 hypomethylation led to genotype-specific alterations of the trichome pattern

We then tested if H3K4me1 maintains robust phenotypes by buffering against different genetic backgrounds. We outcrossed the TrrCA and TrrWT lines (see morphological analysis of larvae and adult flies in the STAR Methods) with three balancer lines, whose lack of recombination could impair the purging of slightly deleterious genetic variants (Rutherford and Lindquist, 1998). We observed increased frequencies of aberrant trichome patterns with H3K4me1 hypomethylation (Figures 2E–2H and S3A). To increase the range of genotypes, we performed crosses with three lines from the Drosophila Genetic Reference Panel (DGRP), which could contain specific standing genetic variations (Mackay et al., 2012). We again found an increased frequency of altered trichome patterns in these genetic backgrounds (Figure 2I–2L and S3A). The affected abdominal segment and the trichome rows that were missing/modified were genotype specific (Figures 2E–2L).

Furthermore, some TrrCA adults from these crosses showed wing defects, with one or both wings crumpled (Figures S3B–S3E). Only crosses between TrrCA and specific genotypes showed higher penetrance of this phenotype compared with TrrWT (Figure S3E), again suggesting a genotype-specific effect of hypomethylation.

Hypomonomethylation of H3K4 led to increased adult and larval size

The prevalence of H3K4me1 across the genome (Rada-Iglesias, 2018) connects it to many active or primed enhancers. This dense connectivity to regulatory networks of different functions may have a pleiotropic influence on complex traits. Thus, to understand the impact of H3K4me1 hypomethylation on these traits, we analyzed phenotypes resulting from multiple interacting regulatory and signaling networks.

TrrCA adult flies were larger than TrrWT ones (average body length: 2.34 mm in TrrWT versus 2.40 mm in TrrCA; Figures S4A–S4C). This is consistent with a previous work showing that *trr* restricted growth in a cell-autonomous manner (Kanda et al., 2013). However, the effects of H3K4me1 hypomethylation on the size of adult body features have not been measured. Adopting a morphometric approach (Mayer, 1943), we measured the length of three features that are employed to distinguish morphs or species (McNamee and Dytham, 1993; Tran et al., 2020): wing intervein length, the tibia length, and head width. All three structures increased in size in hypomethylated flies (average wing intervein length: 171 μm in TrrWT versus 181 μm in TrrCA; average tibia length: 0.49 mm in TrrWT versus 0.52 mm in TrrCA; average head width: 0.79 mm in TrrWT versus 0.84 mm in TrrCA; Figures S4D and S4F–S3H). The difference in thorax size, head width, and wing intervein length (but not tibia length) increased with temperature during development (Figures S4E–S4H).

This effect was not restricted to adult flies: hypomethylated larvae were also larger than control larvae at the same stage of development (average body area: 1.02 mm^2 in TrrWT versus 1.44 mm^2 in TrrCA; Figures 3A–3C). However, pupariation time was not affected (Figures S5A). A possible explanation is that different H3K4me1 levels may alter lipid metabolism, a known regulator of larval size (Texada et al., 2020).

Mass spectrometry of hypomethylated larvae revealed increased triglyceride content

To test if H3K4me1 hypomethylation alters lipid metabolism, we used MALDI imaging mass spectrometry, a technique for spatial lipidomics that can detect lipids with spatial resolution (McDonnell and Heeren, 2007). Larval populations of TrrCA and TrrWT were cryosectioned to 20 μm sections (Figure 3D) and analyzed by MALDI imaging. We used a medium scanning resolution of

CellPress
OPEN ACCESS

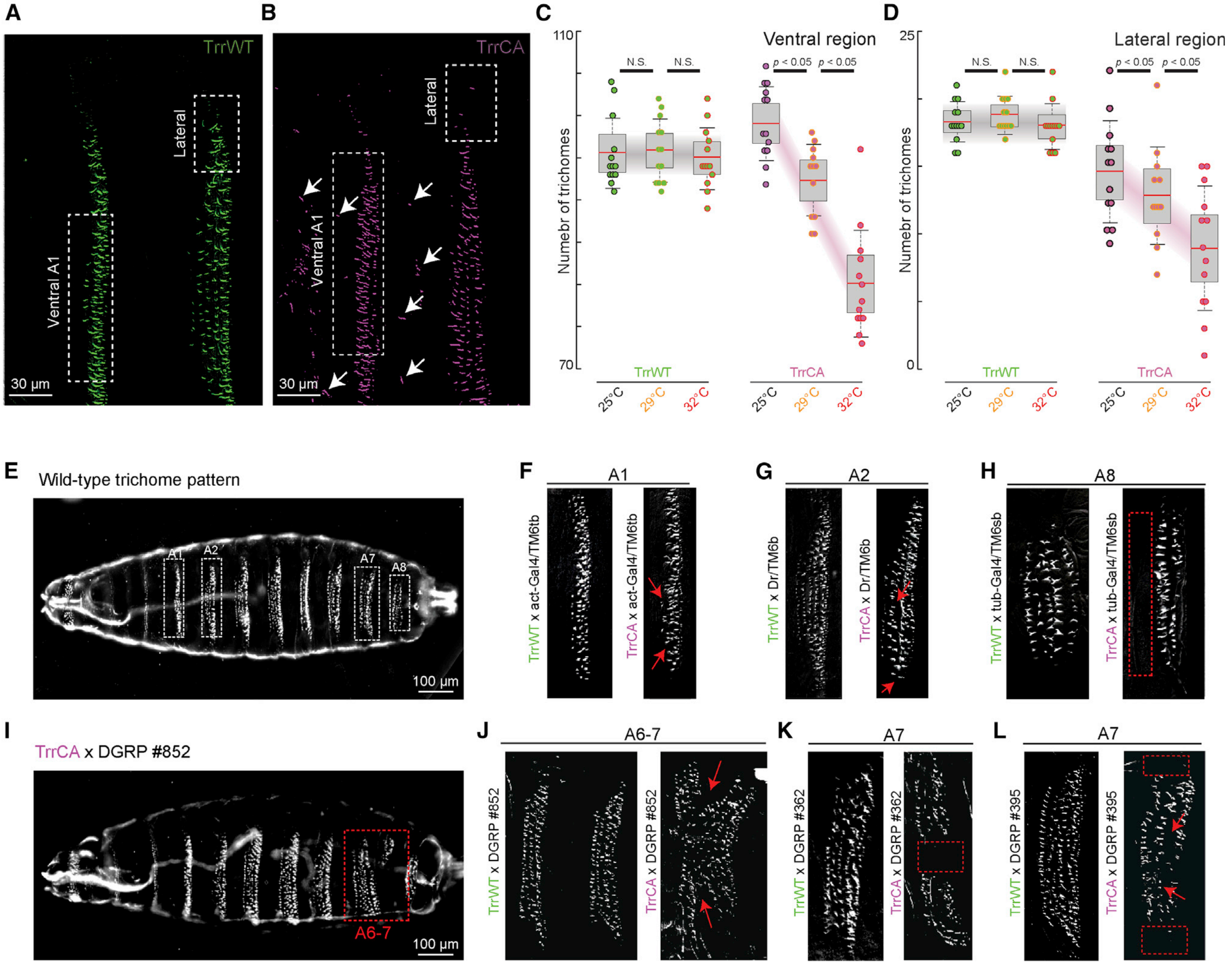

Figure 2. H3K4me1 buffers trichome morphology against deleterious genetic variants

(A and B) Trichome patterns of the first two abdominal segments at 25°C in both trr^1 lines. The white arrows highlight ectopic trichomes.

(C and D) Number of trichomes in the ventral box and the lateral box, respectively, in both trr^1 lines and at different temperatures. Number of larvae quantified: 13 TrrWT and 13 TrrCA at 25°C, 13 TrrWT and 12 TrrCA at 29°C, and 13 TrrWT and 13 TrrCA at 32°C. The center line is the mean. The boxed region is one SD, and the tails are two SDs (95%). p values are from two-tailed Student's t test. N.S., not significant.

(E) WT trichome pattern as observed through dark field microscopy, with boxes highlighting the abdominal segments affected in the crosses.

(F–L) Specific abdominal segments (A1, A2, A6, A7, or A8) of cuticle preparations. The red arrows and dashed boxes highlight the defects. The trr^1 mutant lines were crossed with (F) act-gal4/TM6tb, (G) Dr/TM6b, and (H) tub-Gal4/TM6sb balancer stocks or the (I and J) DGRP #852, (K) DGRP #362, or (L) DGRP #395 lines. Related to Figure S3.

100 μm^2 per pixel to obtain metabolomics data from a population of larvae in the same mass spectrometry run (Figure 3E). In larvae exposed to standard lab food, hypomethylation increased triglyceride levels and reduced glycerophospholipid abundance (Figures 3F–3H and S5B). The elevated triglyceride concentration was confirmed by a biochemical assay (mean triglycerides concentration: 0.318 nmole/larva in TrrWT versus 0.547 nmole/larva in TrrCA; Figures S5C).

We next tested the effect of hypomethylation when larvae were raised using an apple-based medium as a non-optimum, carbohydrate-rich food source. A principal-component analysis (PCA) of single-larva lipidomic profiles integrating intensities of 77 lipids detected across all conditions revealed that the feeding regime altered the effects of H3K4me1 hypomethylation (i.e., apple versus standard; Figures 3I, S5B, S5D, and S5E). In contrast to standard lab food, hypomethylated larvae raised on apples had increased levels of glycerophosphoethanolamines with unaltered triglyceride abundance (Figures S5D). This population-level analysis of lipid signatures suggests that H3K4me1 hypomethylation alters global larval lipid metabolism in a food-dependent manner.

Hypomethylation altered larval behavior on non-standard food sources

Metabolic states can alter behavior in *Drosophila* (Mann et al., 2021). Therefore, we measured the crawling velocity of larvae

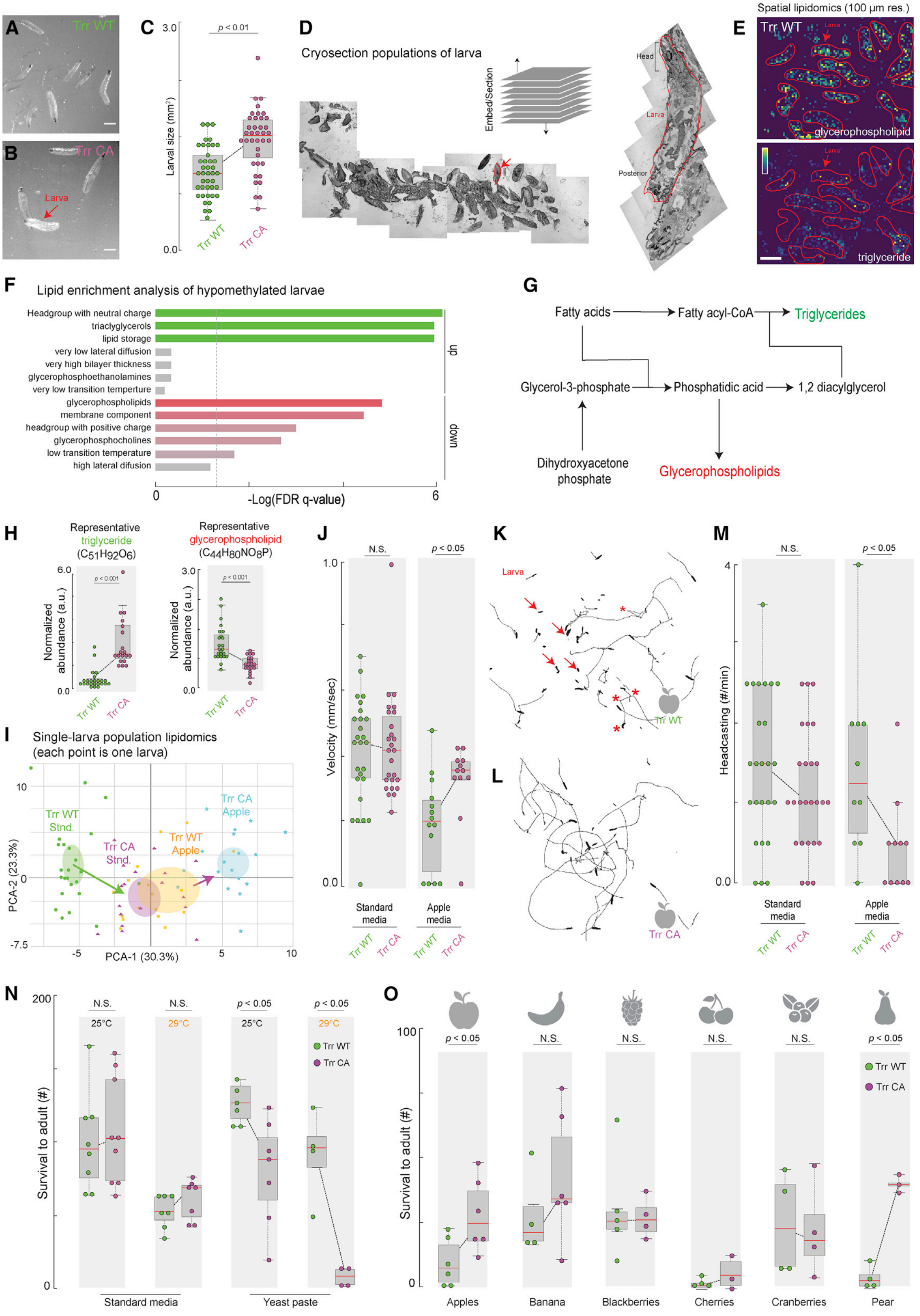

(legend on next page)

from both TrrCA and TrrWT that were developed either on standard lab food or apple-based medium. While both genotypes had similar mean speeds on standard lab food (Figure 3J left), TrrCA larvae on apple-based medium crawled faster than TrrWT larvae (average speed: 0.190 mm/s in TrrWT versus 0.322 mm/s in TrrCA; Figure 3J, right). Moreover, TrrCA larvae had different crawling behaviors on apple food (Figures 3K and 3L). Thus, we quantified the frequency of exploratory head casting, a stereotyped larval behavior (Berni et al., 2012). Similar to crawling velocities, we only found differences on apple-based food (average frequency of head casting: 1.45 events per min in TrrWT versus 0.45 events per min in TrrCA; Figure 3M). In summary, reduction in H3K4me1 changed larval behavior on food sources not commonly used in the laboratory but available in nature.

Hypomethylation altered offspring production in environment-specific manners

Life-history traits are dependent on the metabolic and behavioral profiles of individuals. Thus, we analyzed if H3K4me1 hypomethylation can alter offspring production in different environments, including non-standard feeding regimes. We set up mating groups (20 females and 10 males) from TrrCA or TrrWT lines in vials containing standard lab food, standard lab food supplemented with yeast paste, or several media produced from organically grown fruits, including the apple-based food. The number of eclosed adults after 2 weeks was similar under standard feeding conditions even at 29°C (Figure 3N, left). In contrast, supplementing with yeast paste decreased the offspring number of TrrCA compared with TrrWT. Increasing the temperature to 29°C exacerbated this effect (mean number of eclosed adults: 136.29 for TrrWT versus 79.43 for TrrCA at 25°C, 90.75 for TrrWT versus 7.75 for TrrCA at 29°C; Figure 3N, right). Fruit-based foods had more heterogeneous results. For example, the TrrCA line had increased progeny with apple- and pear-based foods but not with blackberries or cranberries (mean number of eclosed adults: 10 for TrrWT versus 28.5 for TrrCA in apples, 4.25 for TrrWT versus 34.67 for TrrCA in pears; Figure 3O). Although a comprehensive analysis of the chemical composition of these food sources would be required to explain the observed differences in progeny production, these results show that H3K4me1 hypomethylation could both negatively and positively alter offspring production depending on available food sources.

Mutation of the native *trr* locus recapitulates phenotypic alterations

To verify that the observed effects in TrrCA are linked to hypomethylation, we modified the native *trr* locus in w^{1118} with the same mutation as TrrCA (Cys at position 2398 to Ala). This CRISPR.TrrCA line (Figures 4A–4E) showed many of the phenotypes that we detected in the trr^1;TrrCA line (Figures 4A–4E), suggesting that the effects of hypomethylation described here are consistent between populations and genetic background variations.

DISCUSSION

H3K4me1 is a canonical histone modification marking transcriptional enhancers across many genomes (Roy et al., 2010). Despite its ubiquity, H3K4me1 deficiency is tolerated under standard laboratory conditions (Dorighi et al., 2017; Rickels et al., 2017), and gene expression remains mostly unaffected (Rickels et al., 2017). A possible explanation has been that H3K4me1 fine-tunes enhancers, permitting nuanced responses to environmental perturbations (Rickels et al., 2017). Furthermore, chromatin regulators could buffer variations in gene expression, which might be a common characteristics of genome-wide chromatin regulators (Richardson et al., 2013; Tirosh et al., 2010).

Figure 3. H3K4 hypomethylation affects *Drosophila* biology across many phenotypic levels

(A and B) Pictures of 120 h-old larvae from TrrWT (A) or TrrCA (B) (scale bar: 830 μm).

(C) The mean size of TrrCA larvae versus TrrWT (n = 41 for TrrWT and n = 39 for TrrCA).

(D) Top panel: middle section of a larval population. The red arrow highlights an individual larva. Bottom panel: middle section of a single larva at higher magnification.

(E) Medium-resolution MALDI imaging analysis for a larval population (third instar) showing the relative intensities of a glycerophospholipid (top panel) at *m/z* = 744.5537 (C41H78NO8P) and a triglyceride (bottom panel) at *m/z* = 815.6525 (C49H92O6); scale bar: 1 mm.

(F) Enrichment analysis comparing TrrWT versus TrrCA based on the abundance of 77 metabolites detected in all tested conditions (the full list is in Figure S5B).

(G) Schematic of lipid metabolism with triglycerides (green) and glycerophospholipids (red) highlighted.

(H) Abundance of a representative triglyceride (left) and glycerophospholipid (right) obtained by MALDI imaging mass spectrometry (spec) with single-larva resolution. Each dot represents a larva with n = 24 for TrrWT and n = 20 for TrrCA.

(I) Principal-component analysis (PCA) based on single-larva abundance of 77 different lipids identified by MALDI imaging mass spec. Each dot represents a larva. n = 23 for TrrWT standard, n = 20 for TrrCA standard, n = 17 for TrrWT apples, and n = 20 for TrrCA apples.

(J) Average velocity of individual larvae grown on standard lab food or apple-based food. n = 26 for TrrWT and TrrCA on standard food, n = 14 for TrrWT on apples, and n = 12 for TrrCA on apples.

(K and L) Two min trajectories of TrrWT (K) or TrrCA (L) larvae grown on apple-based food. Red arrows point to larvae that remained still throughout the recording. Red stars show path changes associated with head casting.

(M) Frequency of head casting in both trr^1 mutant lines on standard or apple-based food. Only moving larvae were inluded. n = 26 for TrrWT and TrrCA on standard food, n = 14 for TrrWT on apples, and n = 12 for TrrCA on apples.

(N) The number of offspring flies produced by trr^1 mutant lines with an equal number of parents in 2 weeks on standard lab medium (left) or lab food enriched with yeast paste (right) and at 25°C or 29°C. Each dot represents an independent replicate population.

(O) Similar to (N) but carried out in food sources produced from slightly rotten organic fruits.

For all panels in the figure: the center line is the mean. The boxed region is one SD, and the tails are two SDs (95%). p values are from two-tailed Student's t test comparing the two trr^1 lines except for (C), which uses the right-tailed Student's t test; N.S., not significant.

Related to Figures S4 and S5.

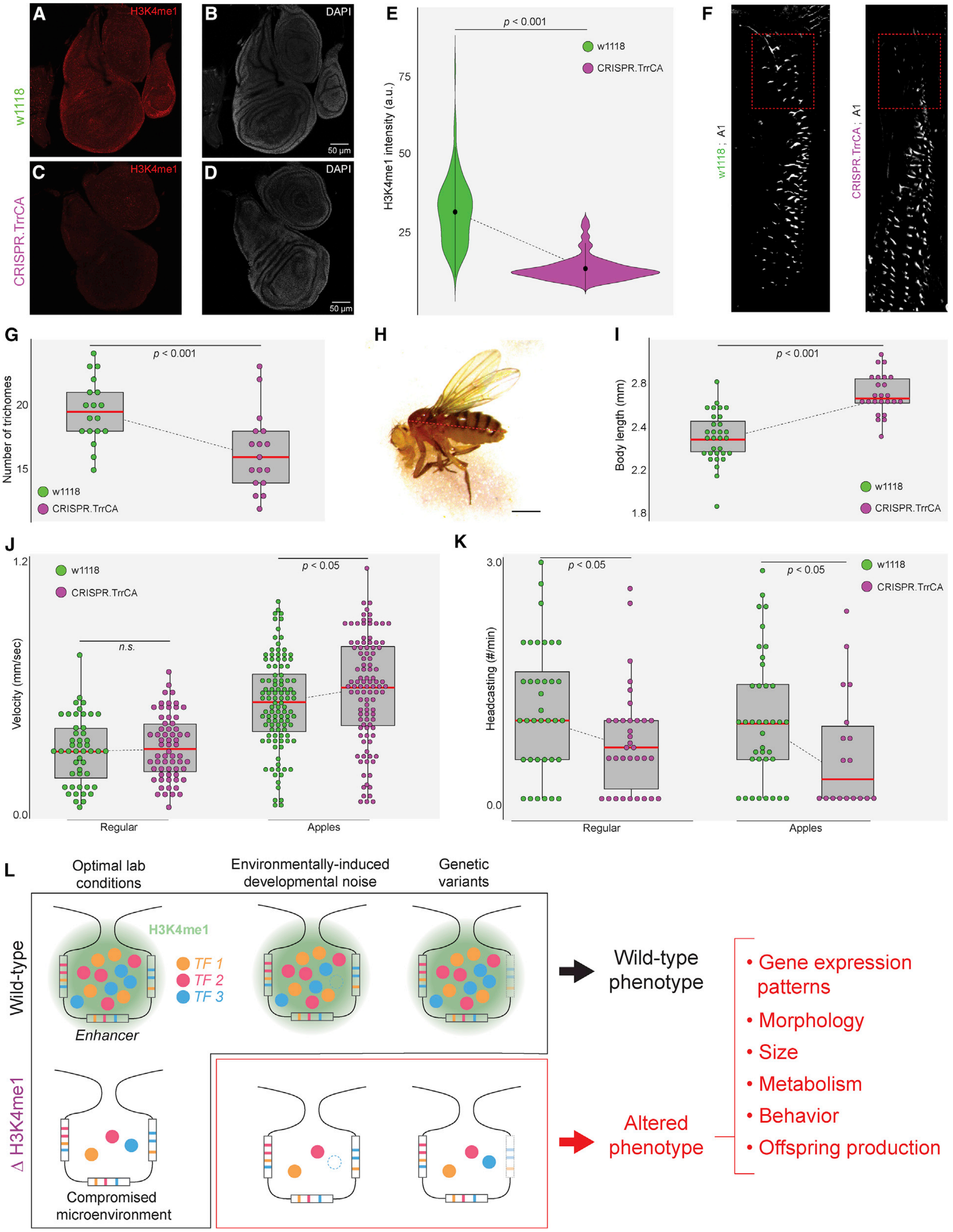

(legend on next page)

Applying a phenomics approach (Houle et al., 2010) across animal development, we acquired phenotypic data that range from gene expression to behavior. We showed that H3K4me1 supports regulatory robustness against environmental and genetic variations (Figure 4L). For individual regulatory networks, it maintains correct gene expression and cell fate determination by supporting transcriptional microenvironments. Thus, H3K4me1, including the associated H3K4me3 (Andersson, 2015), may guide multivalent proteins into transcriptional condensates to activate or silence gene expression (Tsai et al., 2020). Histone marks could stabilize localized protein concentrations and activate them at the proper place and time during development, conferring transcriptional robustness to target genes.

In a population, H3K4me1 conceals genetic variations that could cause unfavorable phenotypes, potentially functioning as a phenotypic capacitor (Rutherford and Lindquist, 1998) (Figure 4L). Notably, H3K4me1 hypomethylation did not completely disrupt any analyzed phenotypes but altered them in specific ways. For example, trichome and wing defects appeared only with certain genetic backgrounds (Figures 2E–2L). Hypomethylation even increased offspring production with certain food sources (Figure 3O). As biological systems rely on multiple elements and processes to develop robust phenotypes (e.g., feedback loops, shadow enhancers, microRNAs [miRNAs], etc.), removing a single histone mark should not collapse the developmental program but instead lead to specific alternative phenotypic states (e.g., higher triglyceride content). This multimodal landscape might be a feature of adaptive evolution, as the tuning of different robustness-conferring mechanisms could lead to the emergence of novel features without compromising viability.

The developmental phenomics workflow introduced here tracked how depleting a histone mark affects the entire biological system. Observed phenotypic changes across development suggest that H3K4me1 fosters robustness through transcriptional microenvironments (Figure 4L). The regulatory mechanisms underlying these microenvironments and their physiological implications are just starting to be explored (Tsai et al., 2020). DNA accessibility is a key element in the clustering of TFs and polymerases in transcriptional hubs (Porcelli et al., 2019); however, evidence for a histone mark supporting nuclear microenvironments had not been reported. Future research should reveal the molecular elements that collaborate with H3K4me1 to establish and maintain nuclear microenvironments. The use of MALDI imaging mass spectrometry for the measurement of lipid profiles at a population level revealed distinct metabolic profiles in larvae that outwardly appeared to be WT. The same approach can detect small molecules, small peptides, glycans, and exogenous molecules such as drugs or pollutants (Hsieh et al., 2007), enabling fast and cost-efficient metabolic phenotyping at a populational scale. Combined with robotics (Fuqua et al., 2020, 2021) and automated behavioral characterization (Berman et al., 2014), our approach could turn phenomics into a standardizable and quantitative phenotyping method for multiple fields of biological research.

In conclusion, this work highlights the risks of stripping away how the environment interacts with the animal genome during development. While under standard laboratory conditions, H3K4me1 appears to be dispensable for development and viability (Rickels et al., 2017); their effects become clear under conditions that approximate what *Drosophila melanogaster* experiences in nature (Figure 3N and 3O). Thus, incorporating realistic environmental contexts into our experimental design is essential for understanding the regulatory genome and its contribution to evolution and development (Bergelson et al., 2021). In the future, phenomics and the inclusion of ecologically relevant conditions should explore how modulating elements embedded in densely connected biological networks could lead to the emergence of novel traits and influence the evolutionary dynamics of populations (Alvarez-Ponce et al., 2017; Jovelin and Phillips, 2009; Yamada and Bork, 2009).

Limitations of the study

We have shown here that H3K4me1 supports the recruitment of Ubx to *svb* transcription sites, thus contributing to the establishment of nuclear microenvironments that preserve transcriptional output and trichome development. Even though generalizing this mechanism to other regulatory networks could explain the phenotypic changes we observed, direct evidence is necessary to confirm this. Further research should confirm that the

Figure 4. H3K4me1 buffers phenotype development against environmental and genetic perturbations

(A–D) Reduction in H3K4me1 in a CRISPR-built TrrCA allele (CRISPR.TrrCA) at the native locus. (A and C) Immunostaining for H3K4me1 or (B and D) DAPI staining for DNA in wing discs of w^{1118} or CRISPR.TrrCA.

(E) Single-cell intensity of the H3K4me1 signal in wing discs of w^{1118} or CRISPR.TrrCA (n = 125, from 10 different wing imaginal discs per genotype). The dot is the mean, and the bar is two SDs.

(F) Trichome pattern in the first abdominal segment at 29°C in w^{1118} or the CRISPR.TrrCA line. The dashed box highlights the lateral region where the number of trichomes was counted.

(G) Number of trichomes in the lateral box in w^{1118} and CRISPR.TrrCA at 29°C. Number of larvae quantified: 18 for w^{1118} and 17 for CRISPR.TrrCA.

(H) Pictures of 72 h-old (after emergence) adult flies from CRISPR.TrrCA (scale bar: 0.5 mm). The dashed lines show the measured body length.

(I) The body length of CRISPR.TrrCA flies versus w^{1118} (n = 31 for w^{1118} and n = 24 for CRISPR.TrrCA).

(J) Average velocity of individual larvae grown on standard lab food or apple-based food.

(K) Frequency of head casting on standard or apple-based food. Only moving larvae were measured.

(L) Biological role of H3K4me1 as revealed by the trr^1 mutant and the CRISPR.TrrCA lines. H3K4me1 supports transcriptional microenvironments, where the local clustering of TFs and enhancers buffer against the effect of environmental and genetic perturbations on transcriptional output. The absence of H3K4me1 alters phenotypes at multiple levels, leading to context-dependent alterations in size, morphology, metabolism, behavior, and adaptability.

For all panels in the figure: center line, mean; upper and lower limits, SD; whiskers, 2 SDs, 95% confidence intervals (CIs). Two-tailed Student's t test comparing the two trr^1 lines; N.S., not significant.

Related to Table S1.

metabolic, behavioral, or fitness-related changes in hypomethylated populations are indeed derived from compromised nuclear microenvironments. Furthermore, in both the *trr*[1];TrrCA line and the TrrCA.CRISPR line, the levels of H3K4me1 are reduced across the entire genome. Additional tools must be developed in order to address the effect of H3K4me1 hypomethylation in specific enhancers or gene regulatory networks. Finally, this study is restricted to the analysis of genetically induced hypomethylation. Future research should address the standing variation of global H3K4me1 patterns and at the *trr* locus in wild *Drosophila* populations.

STAR★METHODS

Detailed methods are provided in the online version of this paper and include the following:

- KEY RESOURCES TABLE
- RESOURCE AVAILABILITY
  - Lead contact
  - Materials availability
  - Data and code availability
- EXPERIMENTAL MODEL AND SUBJECT DETAILS
- METHOD DETAILS
  - H3K4me1 ChIP-Seq
  - Correlation between H3K4me1 deposition and the regulation of the *svb* network
  - Sample preparation and staining for confocal imaging
  - Primary antibodies
  - Secondary antibodies
  - Morphological analysis of larvae and adult flies
  - Offspring production assay with different temperatures and food sources
  - Larval lipidomics assays with MALDI-imaging
  - Triglycerides quantification assay
  - Larval behavioral assays
  - New TrrCA allele developed with CRISPR/Cas9
- QUANTIFICATION AND STATISTICAL ANALYSIS
  - Confocal image acquisition and analysis
  - ChIP-seq data
  - Metabolomics
  - Experimental design

SUPPLEMENTAL INFORMATION

Supplemental information can be found online at https://doi.org/10.1016/j.celrep.2022.111832.

ACKNOWLEDGMENTS

A.T. is supported by the German Research Foundation (Deutsche Forschungsgemeinschaft, grant number TS 428/1-1). M.E. and T.A. are supported by the ERC Consolidator grant METACELL (grant agreement 773089). R.G. and L.G. are supported by fellowships from the European Molecular Biology Laboratory Interdisciplinary Postdoc Programme (EIPOD) under Marie Skłodowska-Curie Actions COFUND (grant agreement numbers 664726 and 847543, respectively). A.T., M.E., T.A., and J.C. are supported by EMBL. E.P.-B.N. is supported by the Israel Science Foundation (grant no. 2567/20). The transgenic fly lines containing Trr methylation mutants were a generous gift from A. Shilatifard.

AUTHOR CONTRIBUTIONS

Conceptualization, L.G., A.T., and J.C.; investigation, L.G., A.T., M.E., R.G., E.P.-B.N., and J.C.; methodology, L.G., A.T., M.E., T.A., and J.C.; formal analysis, L.G., A.T., M.E., R.G., and J.C.; visualization, L.G., A.T., R.G., and J.C.; supervision, A.T., T.A., and J.C.; project administration, J.C.; funding acquisition, A.T., E.P.-B.N., T.A., and J.C.; writing – original draft, L.G., A.T., and J.C.; writing – review & editing, L.G., A.T., M.E., R.G., E.P.-B.N., T.A., and J.C.

DECLARATION OF INTERESTS

The authors declare no competing interests.

INCLUSION AND DIVERSITY

We support inclusive, diverse, and equitable conduct of research.

Received: June 15, 2022
Revised: August 29, 2022
Accepted: November 22, 2022
Published: December 13, 2022

REFERENCES

Alexandrov, T., Ovchinnikova, K., Palmer, A., Kovalev, V., Tarasov, A., Stuart, L., Nigmetzianov, R., Fay, D., METASPACE contributors, K., Gaudin, M., et al. (2019). METASPACE: a community-populated knowledge base of spatial metabolomes in health and disease. Preprint at bioRxiv, 539478. https://doi.org/10.1101/539478.

Alvarez-Ponce, D., Feyertag, F., and Chakraborty, S. (2017). Position matters: network centrality considerably impacts rates of protein evolution in the human protein–protein interaction network. Genome Biol. Evol. *9*, 1742–1756. https://doi.org/10.1093/gbe/evx117.

Andersson, R. (2015). Promoter or enhancer, what's the difference? Deconstruction of established distinctions and presentation of a unifying model. Bioessays *37*, 314–323. https://doi.org/10.1002/bies.201400162.

Barski, A., Cuddapah, S., Cui, K., Roh, T.-Y., Schones, D.E., Wang, Z., Wei, G., Chepelev, I., and Zhao, K. (2007). High-resolution profiling of histone methylations in the human genome. Cell *129*, 823–837. https://doi.org/10.1016/j.cell.2007.05.009.

Bergelson, J., Kreitman, M., Petrov, D.A., Sanchez, A., and Tikhonov, M. (2021). Functional biology in its natural context: a search for emergent simplicity. Elife *10*, e67646. https://doi.org/10.7554/ELIFE.67646.

Berman, G.J., Choi, D.M., Bialek, W., and Shaevitz, J.W. (2014). Mapping the stereotyped behaviour of freely moving fruit flies. J. R. Soc. Interface *11*, 20140672. https://doi.org/10.1098/rsif.2014.0672.

Berni, J., Pulver, S.R., Griffith, L.C., and Bate, M. (2012). Autonomous circuitry for substrate exploration in freely moving Drosophila larvae. Curr. Biol. *22*, 1861–1870. https://doi.org/10.1016/j.cub.2012.07.048.

Bleckwehl, T., Crispatzu, G., Schaaf, K., Respuela, P., Bartusel, M., Benson, L., Clark, S.J., Dorighi, K.M., Barral, A., Laugsch, M., et al. (2021). Enhancer-associated H3K4 methylation safeguards in vitro germline competence. Nat. Commun. *12*, 5771. https://doi.org/10.1038/s41467-021-26065-6.

Bogdanović, O., Fernandez-Miñán, A., Tena, J.J., de la Calle-Mustienes, E., Hidalgo, C., van Kruysbergen, I., van Heeringen, S.J., Veenstra, G.J.C., Gómez-Skarmeta, J.L., O, B., et al. (2012). Dynamics of enhancer chromatin signatures mark the transition from pluripotency to cell specification during embryogenesis. Genome Res. *22*, 2043–2053. https://doi.org/10.1101/GR.134833.111.

Bonn, S., Zinzen, R.P., Girardot, C., Gustafson, E.H., Perez-Gonzalez, A., Delhomme, N., Ghavi-Helm, Y., Wilczyński, B., Riddell, A., and Furlong, E.E.M. (2012). Tissue-specific analysis of chromatin state identifies temporal signatures of enhancer activity during embryonic development. Nat. Genet. *44*, 148–156. https://doi.org/10.1038/ng.1064.

Chhabra, R., Kolli, S., and Bauer, J.H. (2013). Organically grown food provides health benefits to Drosophila melanogaster. PLoS One *8*, e52988. https://doi.org/10.1371/JOURNAL.PONE.0052988.

Crocker, J., Abe, N., Rinaldi, L., McGregor, A.P., Frankel, N., Wang, S., Alsawadi, A., Valenti, P., Plaza, S., Payre, F., et al. (2015). Low affinity binding site clusters confer hox specificity and regulatory robustness. Cell *160*, 191–203. https://doi.org/10.1016/j.cell.2014.11.041.

Davidson, E.H., and Levine, M.S. (2008). Properties of developmental gene regulatory networks. Proc. Natl. Acad. Sci. USA *105*, 20063–20066, 20063–20066. https://doi.org/10.1073/pnas.0806007105.

Dorighi, K.M., Swigut, T., Henriques, T., Bhanu, N.V., Scruggs, B.S., Nady, N., Still, C.D., Garcia, B.A., Adelman, K., and Wysocka, J. (2017). Mll3 and Mll4 facilitate enhancer RNA synthesis and transcription from promoters independently of H3K4 monomethylation. Mol. Cell *66*, 568–576.e4. https://doi.org/10.1016/j.molcel.2017.04.018.

Frankel, N., Davis, G.K., Vargas, D., Wang, S., Payre, F., and Stern, D.L. (2010). Phenotypic robustness conferred by apparently redundant transcriptional enhancers. Nature *466*, 490–493. https://doi.org/10.1038/nature09158.

Fuqua, T., Jordan, J., van Breugel, M.E., Halavatyi, A., Tischer, C., Polidoro, P., Abe, N., Tsai, A., Mann, R.S., Stern, D.L., and Crocker, J. (2020). Dense and pleiotropic regulatory information in a developmental enhancer. Nature *587*, 235–239. https://doi.org/10.1038/s41586-020-2816-5.

Fuqua, T., Jordan, J., Halavatyi, A., Tischer, C., Richter, K., and Crocker, J. (2021). An open-source semi-automated robotics pipeline for embryo immunohistochemistry. Sci. Rep. *11*, 10314–10316, 2021. https://doi.org/10.1038/s41598-021-89676-5.

Gibert, J.-M., Mouchel-Vielh, E., De Castro, S., and Peronnet, F. (2016). Phenotypic plasticity through transcriptional regulation of the evolutionary hotspot gene tan in Drosophila melanogaster. PLoS Genet. *12*, e1006218. https://doi.org/10.1371/JOURNAL.PGEN.1006218.

Heintzman, N.D., Hon, G.C., Hawkins, R.D., Kheradpour, P., Stark, A., Harp, L.F., Ye, Z., Lee, L.K., Stuart, R.K., Ching, C.W., et al. (2009). Histone modifications at human enhancers reflect global cell-type-specific gene expression. Nature *459*, 108–112. https://doi.org/10.1038/nature07829.

Herz, H.-M., Mohan, M., Garruss, A.S., Liang, K., Takahashi, Y.H., Mickey, K., Voets, O., Verrijzer, C.P., and Shilatifard, A. (2012). Enhancer-associated H3K4 monomethylation by Trithorax-related, the *Drosophila* homolog of mammalian Mll3/Mll4. Genes Dev. *26*, 2604–2620. https://doi.org/10.1101/gad.201327.112.

Houle, D., Govindaraju, D.R., and Omholt, S. (2010). Phenomics: the next challenge. Nat. Rev. Genet. *11*, 855–866. https://doi.org/10.1038/nrg2897.

Hsieh, Y., Chen, J., and Korfmacher, W.A. (2007). Mapping pharmaceuticals in tissues using MALDI imaging mass spectrometry. J. Pharmacol. Toxicol. Methods *55*, 193–200. https://doi.org/10.1016/j.vascn.2006.06.004.

Johnson, W.E., Li, C., and Rabinovic, A. (2007). Adjusting batch effects in microarray expression data using empirical Bayes methods. Biostatistics *8*, 118–127. https://doi.org/10.1093/BIOSTATISTICS/KXJ037.

Jovelin, R., and Phillips, P.C. (2009). Evolutionary rates and centrality in the yeast gene regulatory network. Genome Biol. *10*, R35. https://doi.org/10.1186/gb-2009-10-4-r35.

Kanda, H., Nguyen, A., Chen, L., Okano, H., and Hariharan, I.K. (2013). The Drosophila ortholog of MLL3 and MLL4, trithorax related, functions as a negative regulator of tissue growth. Mol. Cell Biol. *33*, 1702–1710. https://doi.org/10.1128/MCB.01585-12.

Langmead, B., and Salzberg, S.L. (2012). Fast gapped-read alignment with Bowtie 2. Nat. Methods *9*, 357–359, 2012. https://doi.org/10.1038/nmeth.1923.

Lee, J.-E., Wang, C., Xu, S., Cho, Y.-W., Wang, L., Feng, X., Baldridge, A., Sartorelli, V., Zhuang, L., Peng, W., and Ge, K. (2013). H3K4 mono- and di-methyltransferase MLL4 is required for enhancer activation during cell differentiation. Elife *2*, e01503. https://doi.org/10.7554/eLife.01503.

Mackay, T.F.C., Richards, S., Stone, E.A., Barbadilla, A., Ayroles, J.F., Zhu, D., Casillas, S., Han, Y., Magwire, M.M., Cridland, J.M., et al. (2012). The Drosophila melanogaster genetic reference panel. Nature *482*, 173–178. https://doi.org/10.1038/nature10811.

Mann, K., Deny, S., Ganguli, S., and Clandinin, T.R. (2021). Coupling of activity, metabolism and behaviour across the Drosophila brain. Nature *593*, 244–248.

Masel, J., and Siegal, M.L. (2009). Robustness: mechanisms and consequences. Trends Genet. *25*, 395–403. https://doi.org/10.1016/j.tig.2009.07.005.

Mayer, E. (1943). On growth and form. By D'Arcy Wentworth Thompson. A new edition. Cambridge and New York, University Press and Macmillan, 1942, 1116 pp., 554 illustrations, 21½ cm Price, $12.50. Anat. Rec. *85*, 111–116. https://doi.org/10.1002/AR.1090850108.

McDonnell, L.A., and Heeren, R.M.A. (2007). Imaging mass spectrometry. Mass Spectrom. Rev. *26*, 606–643. https://doi.org/10.1002/mas.20124.

McNamee, S., and Dytham, C. (1993). Morphometric discrimination of the sibling species Drosophila melanogaster (meigen) and D. simulans (sturtevant) (Diptera: drosophilidae). System. Entomol. *18*, 231–236. https://doi.org/10.1111/J.1365-3113.1993.TB00663.X.

Mohan, M., Herz, H.-M., Smith, E.R., Zhang, Y., Jackson, J., Washburn, M.P., Florens, L., Eissenberg, J.C., and Shilatifard, A. (2011). The COMPASS family of H3K4 methylases in Drosophila. Mol. Cell Biol. *31*, 4310–4318. https://doi.org/10.1128/MCB.06092-11.

Molenaar, M.R., Jeucken, A., Wassenaar, T.A., van de Lest, C.H.A., Brouwers, J.F., and Helms, J.B. (2019). LION/web: a web-based ontology enrichment tool for lipidomic data analysis. GigaScience *8*, giz061–10. https://doi.org/10.1093/GIGASCIENCE/GIZ061.

Nègre, N., Brown, C.D., Ma, L., Bristow, C.A., Miller, S.W., Wagner, U., Kheradpour, P., Eaton, M.L., Loriaux, P., Sealfon, R., et al. (2011). A cis-regulatory map of the Drosophila genome. Nature *471*, 527–531, 2011. https://doi.org/10.1038/nature09990.

Porcelli, D., Fischer, B., Russell, S., and White, R. (2019). Chromatin accessibility plays a key role in selective targeting of Hox proteins. Genome Biol. *20*, 115. https://doi.org/10.1186/s13059-019-1721-4.

Rada-Iglesias, A. (2018). Is H3K4me1 at enhancers correlative or causative? Nat. Genet. *50*, 4–5. https://doi.org/10.1038/s41588-017-0018-3.

Richardson, J.B., Uppendahl, L.D., Traficante, M.K., Levy, S.F., and Siegal, M.L. (2013). Histone variant HTZ1 shows Extensive Epistasis with, but does not increase robustness to, new mutations. PLoS Genet. *9*, e1003733. https://doi.org/10.1371/journal.pgen.1003733.

Rickels, R., Herz, H.M., Sze, C.C., Cao, K., Morgan, M.A., Collings, C.K., Gause, M., Takahashi, Y.H., Wang, L., Rendleman, E.J., et al. (2017). Histone H3K4 monomethylation catalyzed by Trr and mammalian COMPASS-like proteins at enhancers is dispensable for development and viability. Nat. Genet. *49*, 1647–1653. https://doi.org/10.1038/ng.3965.

modENCODE Consortium; Roy, S., Ernst, J., Kharchenko, P.V., Kheradpour, P., Negre, N., Eaton, M.L., Landolin, J.M., Bristow, C.A., Ma, L., et al. (2010). Identification of functional elements and regulatory circuits by Drosophila modENCODE. Science *330*, 1787–1797. https://doi.org/10.1126/science.1198374.

Rutherford, S.L., and Lindquist, S. (1998). Hsp90 as a capacitor for morphological evolution. Nature *396*, 336–342. https://doi.org/10.1038/24550.

Scheiner, S.M. (2002). Selection experiments and the study of phenotypic plasticity1. J. Evol. Biol. *15*, 889–898. https://doi.org/10.1046/J.1420-9101.2002.00468.X.

Schindelin, J., Arganda-Carreras, I., Frise, E., Kaynig, V., Longair, M., Pietzsch, T., Preibisch, S., Rueden, C., Saalfeld, S., Schmid, B., et al. (2012). Fiji: an open-source platform for biological-image analysis. Nat. Methods *9*, 676–682. https://doi.org/10.1038/nmeth.2019.

Schmid, B., Schindelin, J., Cardona, A., Longair, M., and Heisenberg, M. (2010). A high-level 3D visualization API for Java and ImageJ. BMC Bioinf. *11*, 274. https://doi.org/10.1186/1471-2105-11-274.

Stern, D.L., and Sucena, E. (2011). Preparation of cuticles from unhatched first-instar Drosophila larvaepdb.prot065532. Cold Spring Harb. Protoc. *2011*, pdb.prot065532-pdb.prot065532. https://doi.org/10.1101/pdb.prot065532.

Texada, M.J., Koyama, T., and Rewitz, K. (2020). Regulation of body size and growth control. Genetics *216*, 269–313. https://doi.org/10.1534/GENETICS.120.303095.

Tirosh, I., Reikhav, S., Sigal, N., Assia, Y., and Barkai, N. (2010). Chromatin regulators as capacitors of interspecies variations in gene expression. Mol. Syst. Biol. *6*, 435. https://doi.org/10.1038/msb.2010.84.

Tran, A.K., Hutchison, W.D., and Asplen, M.K. (2020). Morphometric criteria to differentiate Drosophila suzukii (Diptera: Drosophilidae) seasonal morphs. PLoS One *15*, e0228780. https://doi.org/10.1371/JOURNAL.PONE.0228780.

Tsai, A., and Crocker, J. (2022). Nuclear morphogenesis: forming a heterogeneous nucleus during embryogenesis. Development *149*. https://doi.org/10.1242/dev.200266.

Tsai, A., Muthusamy, A.K., Alves, M.R., Lavis, L.D., Singer, R.H., Stern, D.L., and Crocker, J. (2017). Nuclear microenvironments modulate transcription from low-affinity enhancers. Elife *6*, e28975. https://doi.org/10.7554/eLife.28975.

Tsai, A., Alves, M.R., and Crocker, J. (2019). Multi-enhancer transcriptional hubs confer phenotypic robustness. Elife *8*, e45325. https://doi.org/10.7554/eLife.45325.

Tsai, A., Galupa, R., and Crocker, J. (2020). Robust and efficient gene regulation through localized nuclear microenvironments. Development *147*. https://doi.org/10.1242/dev.161430.

Xie, G., Lee, J.-E., McKernan, K., Park, Y.-K., Jang, Y., Liu, C., Peng, W., and Ge, K. (2020). MLL3/MLL4 methyltransferase activities regulate embryonic stem cell differentiation independent of enhancer H3K4me1. Preprint at bioRxiv. https://doi.org/10.1101/2020.09.14.296558.

Yamada, T., and Bork, P. (2009). Evolution of biomolecular networks — lessons from metabolic and protein interactions. Nat. Rev. Mol. Cell Biol. *10*, 791–803. https://doi.org/10.1038/nrm2787.

STAR★METHODS

KEY RESOURCES TABLE

REAGENT or RESOURCE	SOURCE	IDENTIFIER
Antibodies		
Rabbit anti-H3K4me1	Merck	07-436; RRID: AB_310614
Rabbit anti-H3	Abcam	ab1791; RRID: AB_302613
Mouse anti-Ubx	Developmental Studies Hybridoma Bank	FP3.38-C; RRID: AB_10805300
Sheep anti-DIG	Roche	11,333,089,001; RRID: AB_514496
Donkey anti-mouse Alexa 555	ThermoFisher	A31570; RRID: AB_2536180
Donkey anti-rabbit Alexa 488	ThermoFisher	A21206; RRID: AB_2535792
Donkey anti-rabbit Alexa 555	ThermoFisher	A31572; RRID: AB_162543
Donkey anti-sheep Alexa 488	ThermoFisher	A11015; RRID: AB_141362
Donkey anti-sheep Alexa 633	ThermoFisher	A21100; RRID: AB_2535754
Critical commercial assays		
Triglyceride Quantification Colorimetric Kit	Sigma	# MAK266
Deposited data		
Metabolomics data	METASPACE	834c245c-449f-11ed-89c0-5357adf30217
Imaging files	BioImage Archive	S-BIAD562
ChIP-seq data	ArrayExpress	E-MTAB-12396
Experimental models: Organisms/strains		
Drosophila: w1118: w1118;;	Bloomington Drosophila Stock Center	3605
Drosophila: trrWT: trr1;;trr(WT)	Ali Shilatifard's lab	N/A
Drosophila: trrCA: trr1;;trr(C2398A)	Ali Shilatifard's lab	N/A
Drosophila: Dr/TM6:*;;Dr/TM6b*	Bloomington Drosophila Stock Center	BS00211
Drosophila: tub-gal4/TM6:*;;iso tub-Gal4 (VII)/TM6sb*	Maria Leptin's lab	N/A
Drosophila: act-gal4/TM6:*;;act-Gal4/TM6tb*	Bloomington Drosophila Stock Center	3954
Drosophila: DGRP #362	Drosophila Genetic Reference Panel	#362
Drosophila: DGRP #395	Drosophila Genetic Reference Panel	#395
Drosophila: DGRP #852	Drosophila Genetic Reference Panel	#852
Drosophila: VK00027: w[1118]; PBac{y[+mDint2] GFP[E.3xP3] = vas-Cas9} VK00027	Bloomington Drosophila Stock Center	51,324
Drosophila: CRISPR.trrCA: trr(C2398A);;	This manuscript	N/A
Software and algorithms		
FIMTrack v2.1	University of Münster - Computer Vision & Machine Learning Systems	https://www.uni-muenster.de/Geoinformatics.cvmls/media/fim-media.html
FIJI	Fiji is supported by several laboratories and institutions	https://imagej.net/software/fiji/
MATLAB	MathWorks	mathworks.com

RESOURCE AVAILABILITY

Lead contact

Further information and requests for resources and reagents should be directed to and will be fulfilled by the lead contact, Justin Crocker (justin.crocker@embl.de).

Materials availability

The new fly line generated in this study (CRISPR.trrCA) will be available upon reasonable compensation by the requestor for its processing and shipping.

Data and code availability

- Imaging data (cuticle preparations, confocal images, stereoscope images of adult flies, and videos of moving larvae, organized into zip files) have been deposited at the BioImage Archive (BIA, https://www.ebi.ac.uk/bioimage-archive) and are publicly available as of the date of publication. Accession numbers are listed in the key resources table.
- Metabolomics and ChIP-seq data have been deposited at METASPACE (https://metaspace2020.eu/) and ArrayExpress (https://www.ebi.ac.uk/biostudies/arrayexpress), respectively, and are publicly available as of the date of publication. Accession numbers are listed in the key resources table.
- This paper does not report original code.
- Any additional information required to reanalyze the data reported in this paper is available from the lead contact upon request.

EXPERIMENTAL MODEL AND SUBJECT DETAILS

We used w^{1118} as the "wild-type" reference in the experiments shown in Figures 1A, 1B, S1B–S1E, and 4A–4K. Otherwise, we used lines with non-functional Trithorax-related allele (*trr*^{1}) with two different Trr rescue constructs on the third chromosome: the wild-type rescue line (*trr*^{1}*;;trr*(WT)) or the hypomethylated line (*trr*^{1}*;;trr*(C2398A)). In both cases, the rescue constructs include a 12-kb regulatory region which encompasses all known associated regulatory elements. Thus, the expression of these transgenes is supposed to recapitulate the temporal and spatial expression patterns of the endogenous *trr* locus. Integration occurred at the genomic position 89E11(3R). These lines were established and characterized in a previous work (Rickels et al., 2017).

For experiments examining larval and adult phenotypes with different genetic backgrounds, we crossed the *trr*^{1} lines with balancer stocks obtained from the Bloomington Stock center (https://bdsc.indiana.edu/index.html). They are:*;;Dr/TM6b (BS00211)*,*;;iso tub-Gal4 (VII)/TM6sb* (from Maria Leptin) and*;;act-Gal4/TM6tb (3954)*. We also employed lines #362, #395 and #852 from the *Drosophila Genetic Reference Panel* (http://dgrp2.gnets.ncsu.edu/).

All fly strains were kept at standard laboratory conditions at room temperature unless otherwise noted.

METHOD DETAILS

H3K4me1 ChIP-Seq

Stage 15 embryos from a line containing *E10::GFP* and *7::dsRed* transgenes were cross-linked, dissociated and isolated nuclei were immunostained with anti-GFP and anti-dsRed antibodies. Following staining with appropriate secondary antibodies, the E10:GFP and 7:dsRed nuclei, which constitute only 1.6% and 2.1% of the total input material, respectively, were isolated by fluorescence activated cell sorter (FACS, Figure S1B). Chromatin from 250,000 nuclei of each cell sub-populations was isolated and used for ChIP with anti-H3K4me1 and anti-H3 antibodies (abcam) using the iDeal ChIP-seq kit from Diagenode. Libraries were prepared using the Ovation Ultralow V2 DNA-Seq library preparation kit (NuGen) according to the manufacturer instructions. Following sequencing adapters and low-quality reads (<Q20) were trimmed using TrimGalore (http://www.bioinformatics.babraham.ac.uk/projects/trim_galore).

Correlation between H3K4me1 deposition and the regulation of the *svb* network

Segmentation genes with significant H3K4me1 ChIP-seq peaks within 10 kb of the transcription start sites were identified using the modENCODE dataset H3K4me1; Embryos 12–16 h embryonic data (Roy et al., 2010) (ID 780).

Sample preparation and staining for confocal imaging

Embryos for imaging were collected, fixed in 5% PFA for 25 min and stained according to previous protocols (Crocker et al., 2015). To detect *svb* transcription, antisense RNA probes with DIG against the first intron and second exon were made using the primers from (Tsai et al., 2019). For the co-staining of *svb* and H3K4me1 the samples were first immunostained for the histone modification following the immunofluorescence (IF) staining protocol in (Tsai et al., 2017), re-fixed in 5% PFA in PBT (PBS with 0.1% Tween 20) for 20 min, and then stained for *svb* following the FISH protocol (hybridization of the RNA probe, followed by detection using IF staining against DIG), also from (Tsai et al., 2017). For all experiments observing both Ubx and *svb*, we followed the FISH protocol (Tsai et al., 2017) (hybridization of the RNA probe, followed by detection using IF staining against both DIG and Ubx) without an additional re-fixation step.

Primary and secondary antibodies used are listed in the key resources table with the following dilution ratios.

Primary antibodies

Rabbit anti-H3K4me1 (1:250), mouse anti-Ubx (1:20), and sheep anti-DIG: (1:250).

Secondary antibodies

All were used at a dilution ratio of (1:500).

Stained embryo samples were mounted in ProLong Gold + DAPI Mounting Media (Molecular Probes, Eugene, OR) on a glass slide covered with a number 1.5 high precision coverslip.

Morphological analysis of larvae and adult flies

Female virgins from the *trr*[1] mutant lines were crossed with males from different DGRP stocks or balancer lines (see *Fly strains and crosses*). To analyze the larval trichome pattern, these crosses were housed in egg collection chambers. Embryos were then collected from plates and placed in water, on which they developed at 29°C overnight. Afterward, first instar larvae were treated according to standard protocols (Stern and Sucena, 2011) to prepare cuticles for analysis.

Cuticle preps were imaged on a phase-contrast microscope (Zeiss, Germany). The number of trichomes in the A1 ventral band between two sensory cells was counted using a find maximum function in Fiji and reported as "Ventral", as previously described (Tsai et al., 2019). The number of trichomes in the lateral extremity of the ventral band where the *svb* enhancer *DG3* provides exclusive coverage was also counted and reported as "Lateral", as previously described (Frankel et al., 2010; Tsai et al., 2019). As the identification of "ventral" and "lateral" regions is based on morphological landmarks, and thus it is independent of the overall larval size, the method did not need to be adapted for the bigger larval size observed in hypomethylated larvae.

For the morphological analysis of adult flies, these crosses were placed on fresh vials at 29°C. After 16 h of egg laying, adults were removed, and the egg-containing vials were left at 29°C for 10 days. Then, the emerged male adults were anesthetized with CO_2, and wings were analyzed and photographed employing an Olympus stereoscope.

In all crosses, we used the *trr*[1] mutant lines as the female parental strain, to make sure that all the males in the offspring were deprived of a wild-type Trr allele in the endogenous locus. As the female offspring is heterozygous for *trr*[1], and thus it is supposed to have normal H3K4me1 levels, it should be noted that the informed frequencies are likely an underestimation, and should be considered strictly in a qualitative manner.

Offspring production assay with different temperatures and food sources

Populations of 2-day-old flies from the *trr*[1] mutant lines, consisting exactly of 20 females and 10 males, were placed in vials containing standard lab food, standard food supplemented with yeast paste, or food produced from slightly rotten fruits. Bananas and cranberries were purchased from a local store and the rest collected in a local forest (Heidelberg, Germany, GPS Coordinates, 49.38475495291698, 8.71066590019372). After 2 days of egg laying, adults were removed and the vials were placed at 25°C. For lab food and lab food with yeast, replicates were carried out at 29°C. Then, adult offspring were counted in each vial after 14 days.

As standard food, we employed a modified version of the BDSC Cornmeal Food (https://bdsc.indiana.edu/information/recipes/bloomfood.html), consisting of agar 40 g/L, dry yeast 18 g/L, soya powder 10 g/L, corn syrup 22 g/L, malt extract 80 g/L, corn powder 80 g/L, propionic acid 6.25 g/L and Nipagin 2.4 g/L. All fruit-based foods were prepared according to Chhabra et al. (2013) (Chhabra et al., 2013). Briefly, the indicated fruits were homogenized in a blender, and then water was added to a final concentration for the fruit mass of 1.5 g/L. After adding agar (10% m/v), these preparations were heated in a microwave oven and then dispensed into individual vials.

Larval lipidomics assays with MALDI-imaging

Larval tissues were cryo-sectioned before subjecting them to MALDI imaging mass spectrometry. To do this, a small population ($n \approx 10$) of third instar larvae were embedded in a previously heated 5% m/v carboxymethylcellulose (Sigma) solution. After solidification, the obtained molds were sectioned in a Leica CM1950 cryostat at −20C, producing slices with a thickness of 20 μm. These slices were then mounted on regular glass slides, always aiming to preserve the middle section (40–60 μm) of the sectioned larvae.

Uniform coating of tissue sections with microcrystalline matrix material is essential for MALDI-MSI. To process the larval tissues, a 2,5-dihydroxybenzoic acid (DHB) matrix (Sigma Aldrich) 15mg/mL, dissolved in 70% acetonitrile, was applied onto the samples, mounted on regular glass slides, by using a TM-Sprayer robotic sprayer (HTX Technologies, Carrboro, NC, USA). Then, these glass slides containing the larval tissues were mounted onto a custom slide adaptor and loaded into the MS imaging ion source (AP-SMALDI5, TransMIT GmbH, Giessen, Germany). Generated ions were co-axially transferred to a high mass-resolution mass spectrometer (QExactive Plus mass spectrometer, ThermoFisher Scientific). Positive mode MS analysis was carried out in the full scan mode in the mass range of 200–1100 m/z (resolving power R = 140,000 at m/z 200). Metabolite annotation was performed using the METASPACE cloud software (Alexandrov et al., 2019).

Triglycerides quantification assay

The concentration of triglycerides in *Drosophila* larvae was measured using the Triglyceride Quantification Colorimetric Kit from Sigma (Cat. # MAK266). Ten, 120 h old (third instar), larvae from either the TrrWT or TrrCA line were homogenized in an Eppendorf tube on a Nonidet P40 Substitute (Sigma, Cat. # 74385) 5% solution. Then, the triglycerides concentration of each sample was quantified following the instructions provided by the manufacturer. Absorbance was measured at 570 nm. All metabolic determinations were carried out on larvae that came from vials with the same larval density (30 larvae per vial), to avoid effects of crowding on metabolism.

Larval behavioral assays

Larvae from both *trr*[1] mutant lines, either grown in standard lab food or apple-based food, were placed on agar plates, and their movement was recorded using a regular webcam (Logitech, 1080p, 30 Hz) for two minutes. Then, the speed of individual larvae was calculated from their displacement in the x- and y axes, which was obtained using the MTrack2 tracking algorithm (ImageJ). The frequency of head casting for individual larvae was manually

measured in each of these videos.

New TrrCA allele developed with CRISPR/Cas9

We cloned two trr DNA sequences, one upstream and the other downstream to the catalytic domain, to act as RNA guides for CRISPR/Cas9 mediated transgenesis, into the pCFD4 plasmid using FSEI and BBSI. In parallel, we synthesized a trr DNA sequence that includes the above-mentioned guides, but altering the nucleotides that are required to replace a Cys by an Ala at position 2398, to act as template. Silent mutations were also added to prevent Cas9 for recognizing and cutting this new sequence. This new construct was cloned in the pUC57 plasmid. Both construct-containing plasmids were then injected into a fly line that expresses Cas9 exclusively in the female germ line (Bloomington #51324).

Putative transgenic flies were crossed with w^{1118} ones, and sequenced. After multiple crosses with this w^{1118} line to homogenize the genetic background, homozygous TrrCA; lines were established. H3K4me1 levels were tested in this line through immunostaining, on wing imaginal discs from third instar larvae.

The sequences of the three constructs can be found in Table S1.

QUANTIFICATION AND STATISTICAL ANALYSIS

Confocal image acquisition and analysis

Confocal images were acquired on a Zeiss LSM 880 confocal microscope (Zeiss, Germany) under a Zeiss Plan-Apochromat 63x/1.40 NA objective with the appropriate laser lines (405, 488 and/or 561 nm) using the Zeiss-recommended optimal resolution. Imaging processing to locate transcription sites and extract spatial data was performed in Fiji/ImageJ (Schindelin et al., 2012) with native functions and the 3D ImageJ Suite plugin (Schmid et al., 2010). Subsequent data analysis was performed in MATLAB (MathWorks, Natick, MA) to extract transcription site intensity and radial distributions (Tsai et al., 2017, 2019).

ChIP-seq data

Mapping was performed with bowtie2 (Langmead and Salzberg, 2012) using the reference genome dm6 and sensitive end-to-end presets. Unmapped, multi-mapping reads, reads mapping to chrM (and other non-standard chromosomes) and duplicate reads were removed. For normalization, we subtracted bigWig files of H3 ChIP-seq samples from bigWig files of H3K4me1 ChIP-seq samples. For visualization purposes, we averaged normalized replicates (Pearson correlations of 0.86–0.98) and normalized data was smoothened using a moving average smooth of 500bp.

Metabolomics

The Principal Component Analysis of these results was performed on R using the FactoMineR and factoextra packages (http://factominer.free.fr/). Abundance values were batch-corrected using the ComBat method (Johnson et al., 2007). Enrichment analysis were carried out using LION/web (Molenaar et al., 2019).

Experimental design

Sample sizes for most techniques were based on our previous works. For the methods developed in this report, no *a priori* sample size determination was carried out. No data exclusions were performed. When possible, individual larvae were randomly assigned to different experimental conditions (e.g. different food sources). Blinding was used for morphology analysis (Figure 2 and S3). Statistical details for all experiments can be found in the figure legends.

Cell Reports, Volume *41*

Supplemental information

Developmental phenomics suggests that H3K4 monomethylation confers multi-level phenotypic robustness

Lautaro Gandara, Albert Tsai, Måns Ekelöf, Rafael Galupa, Ella Preger-Ben Noon, Theodore Alexandrov, and Justin Crocker

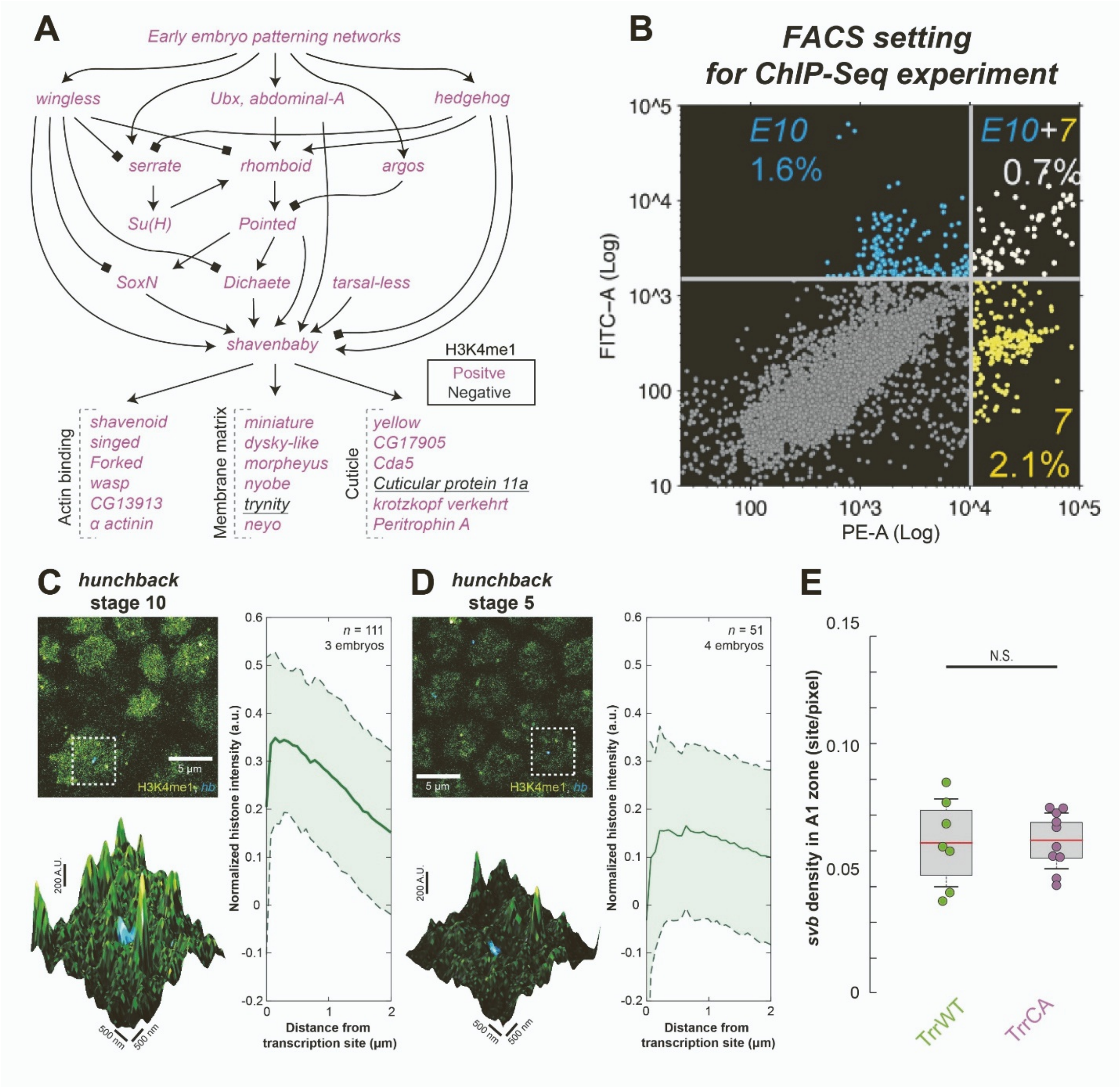

Supplementary Figure 1. H3K4me1 and *svb* transcriptional robustness. Related to Figure 1.

(A) H3K4me1 deposition near developmental transcription factors in the *shavenbaby* regulatory network (the presence of H3K4me1 peaks within 10 kb of the start site for each of the genes in the network is indicated as “positive” and highlighted in magenta).

(B) The setting used to sort cells in FACS for the ChIP-Seq experiment shown in Figure 1B.

(C & D) High resolution confocal imaging experiments in w^{1118} embryos showing the distribution of H3K4me1 in *hb* transcription sites (C for stage 10 and D for stage 5 embryos). The lower panels show a zoomed-in view from the dotted boxes with the height indicating the intensity of the H3K4me1 signal. The normalized average H3K4me1 intensity over multiple transcription sites (C: n=111; D: n=51) is shown on the plots at the right. The shaded region is the variance. C & D were adapted from (Tsai and Crocker, 2022).

(E) Density of *svb* transcription sites in the first abdominal (A1) segment of stage 15 embryos at 25 °C within the ventral band where *svb* is normally expressed (compare with Figure 1H). The number of embryos are: 10 for TrrWT and 7 for TrrCA. The boxed region is one s.d. and the tails are two s.d. (95 %). N.S.: not significant using two-tailed Student's *t*-test

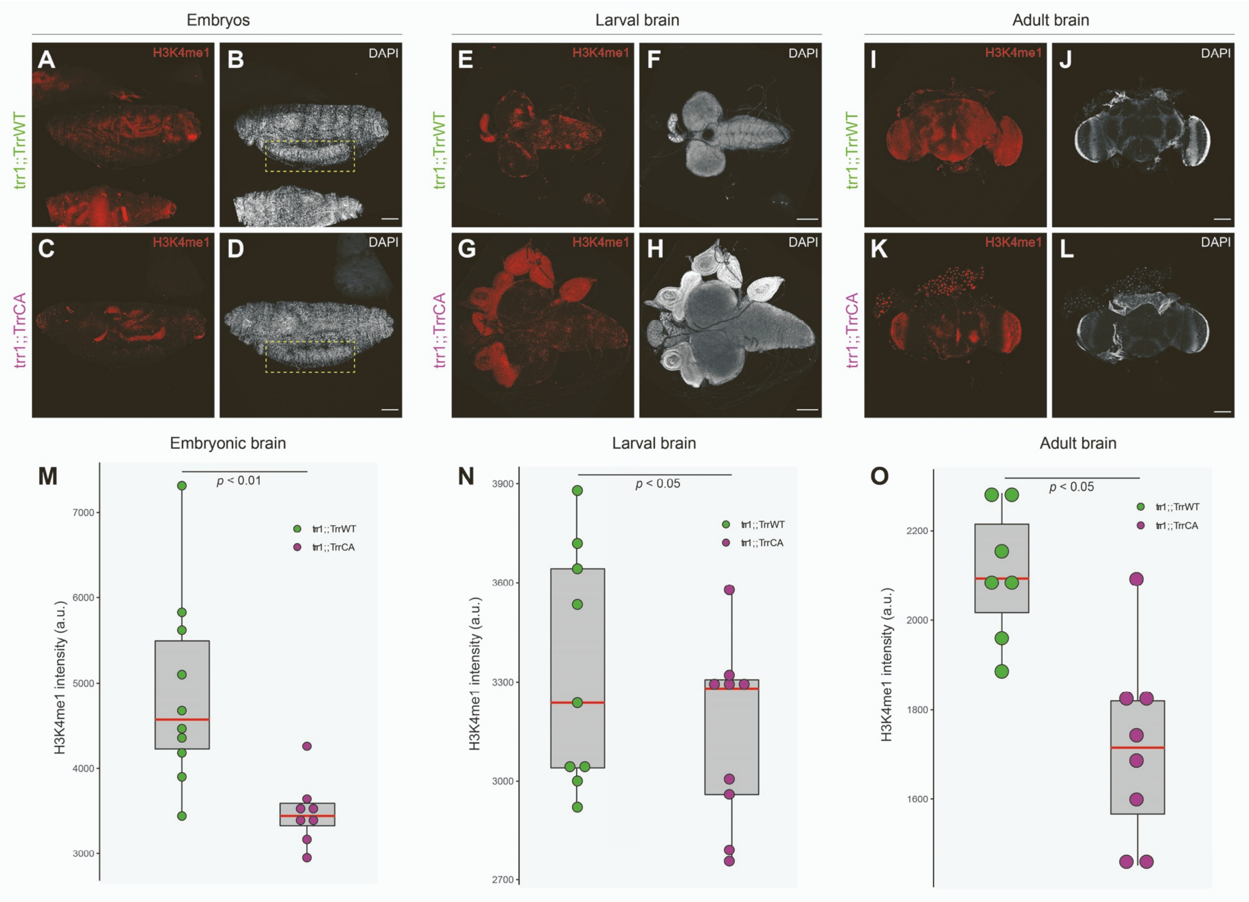

Supplementary Figure 2. H3K4me1 hypomethylation in the central nervous system produced by the TrrCA allele persists throughout the entire life cycle. Related to Figure 1.

(A – L): Immunostaining showing the H3K4me1 signal (A, C, E, G, I & K), or (B, D, F, H, J & L) DAPI staining, in embryonic (A – D, ventral nerve cord is highlighted with yellow dashed boxes), larval (E – H) and adult (I – L) brains. Scale bars: 50 µm in B and D, and 100 µm in F, H, J and L.

(M – O): Intensity of the H3K4me1 signal in embryonic (n=10 for TrrWT and n=8 for TrrCA), larval (n=9 for TrrWT and n=9 for TrrCA) or adult (n=7 for TrrWT and n=8 for TrrCA) brains of *trr1*;;TrrWT or *trr1*;;TrrCA.

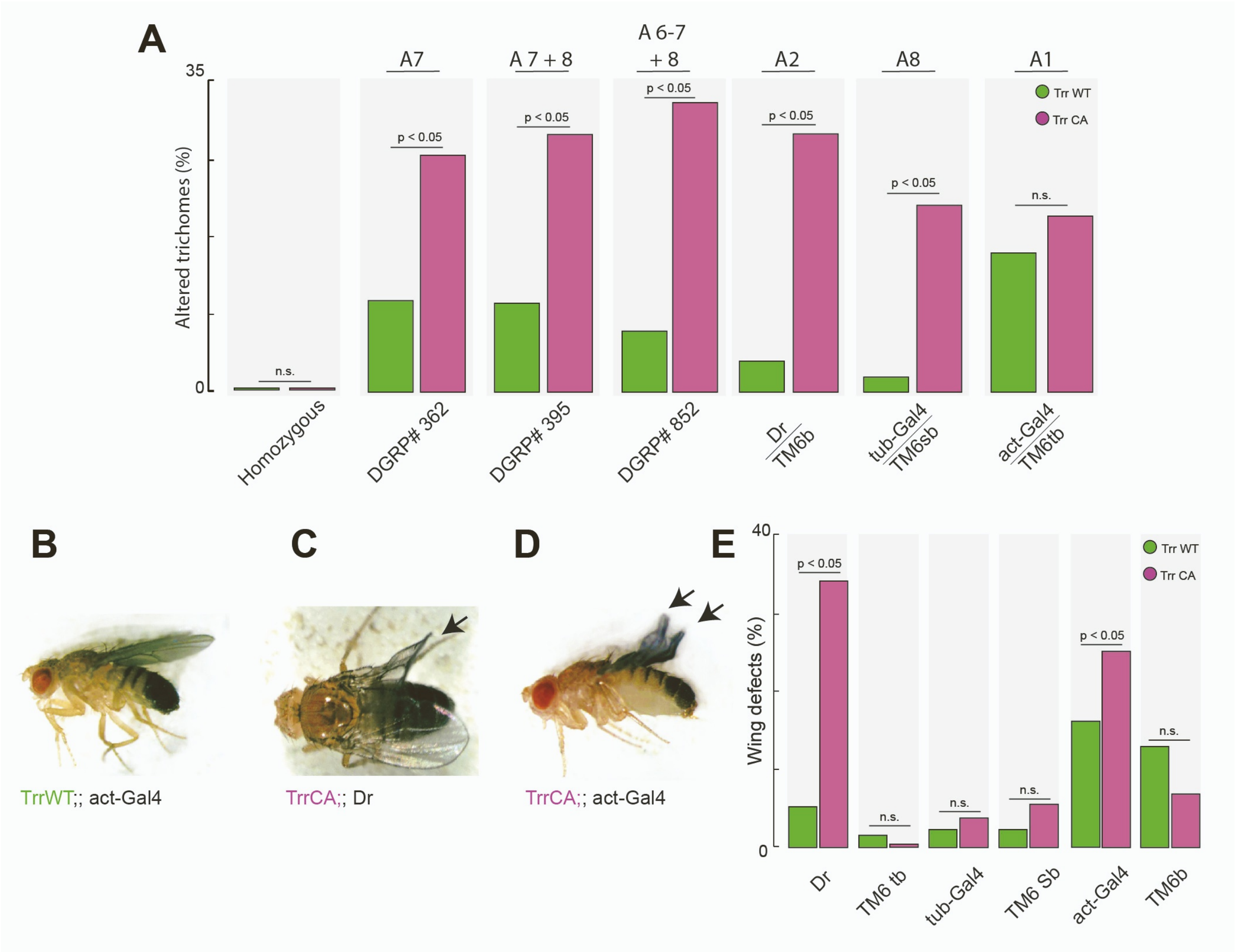

Supplementary Figure 3: Aberrant morphologies in hypomethylated flies with different genetic backgrounds. Related to figure 2.

(A) Frequencies of altered trichome patterns detected in the offspring of the crosses of the *trr*[1] mutant lines with the DGRP lines or balancer stocks. Only the genotype-specific alterations shown in Figure 2E-L were considered for the quantification. n=52 for TrrWT (homozygous), n=35 for TrrCA (homozygous), n=54 for TrrWT x DGRP#362, n=27 for TrrWT x DGRP#395, n=56 for TrrWT x DGRP#852, n=59 for TrrCA x DGRP#362, n=11 for TrrCA x DGRP#395, n=48 for TrrCA x DGRP#852, n=113 for TrrWT x TM6/act-G4, n=156 for TrrWT x TM6/tub-G4, n=45 for TrrWT x TM6/Dr, n=62 for TrrCA x TM6/act-G4, n=51 for TrrCA x TM6/tub-G4, n=57 for TrrCA x TM6/Dr.

(B-D) Pictures of adult *trr^{1}* mutant flies with normal wings from TrrWT, or aberrant wing morphologies (arrows) from TrrCA.

(E) The fraction of flies with deformed wings observed in each of the previously mentioned crosses. n=116 for TrrWT;;act-gal4, n=112 for TrrWT;;TM6 tb, n=152 for TrrWT;;tub-Gal4, n=153 for TrrWT;;TM6 Sb, n=117 for TrrWT;;Dr, n=40 for TrrWT;;TM6b, n=34 for TrrCA;;act-gal4, n=72 for TrrCA;;TM6 tb, n=48 for TrrCA;;tub-Gal4, n=93 for TrrCA;;TM6 Sb, n=19 for TrrCA;;Dr, n=26 for TrrCA;;TM6b

All *p*-values in the figure are from Chi-Square goodness of fit tests. n.s.: not significant.

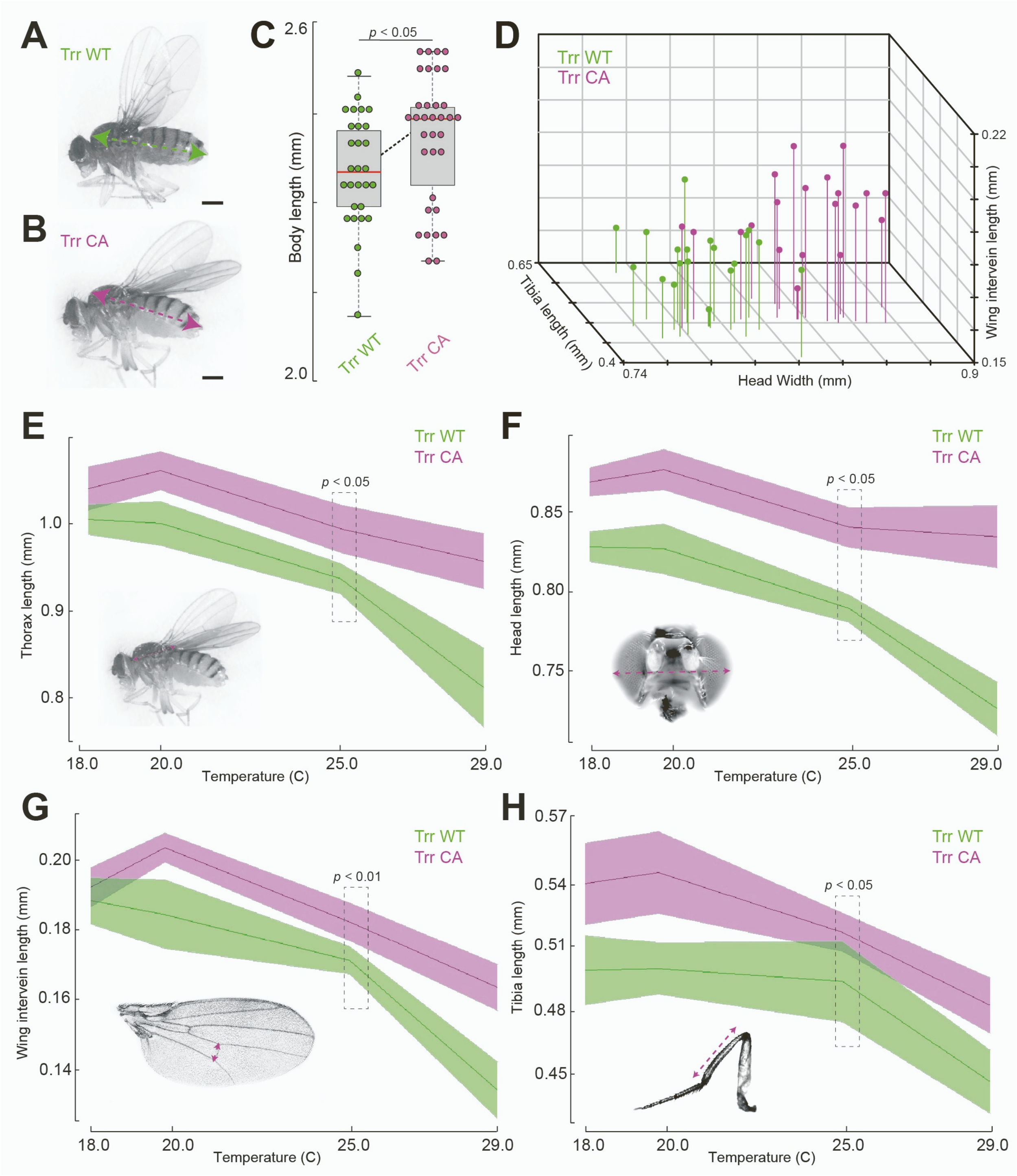

Supplementary Figure 4: H3K4me1 controls body size in adults. Related to Figure 3.

(A & B) Pictures of 72 h old (after emergence) adult flies from TrrWT (A) or TrrCA (B) (scale bar = 0.5 mm). The dashed lines show the measured body length.

(C) The body length of TrrCA flies versus TrrWT (n=29 for TrrWT and n=34 for TrrCA). Center line, mean; upper and lower limits, s.d.; whiskers, 95% CIs.

(D) 3D plot showing the values from panels d to f but linked to single individuals from TrrWT or TrrCA (n=21).

(E - H) Length of the thorax (E), the head width (F), the posterior cross-vein (G), and the fore tibia (H) in both TrrWT and TrrCA adult flies developed at different temperatures(n=20). Center line, mean; upper and lower limits, s.d.

All *p*-values are from two-tailed Student's *t*-test comparing the two trr^1 lines.

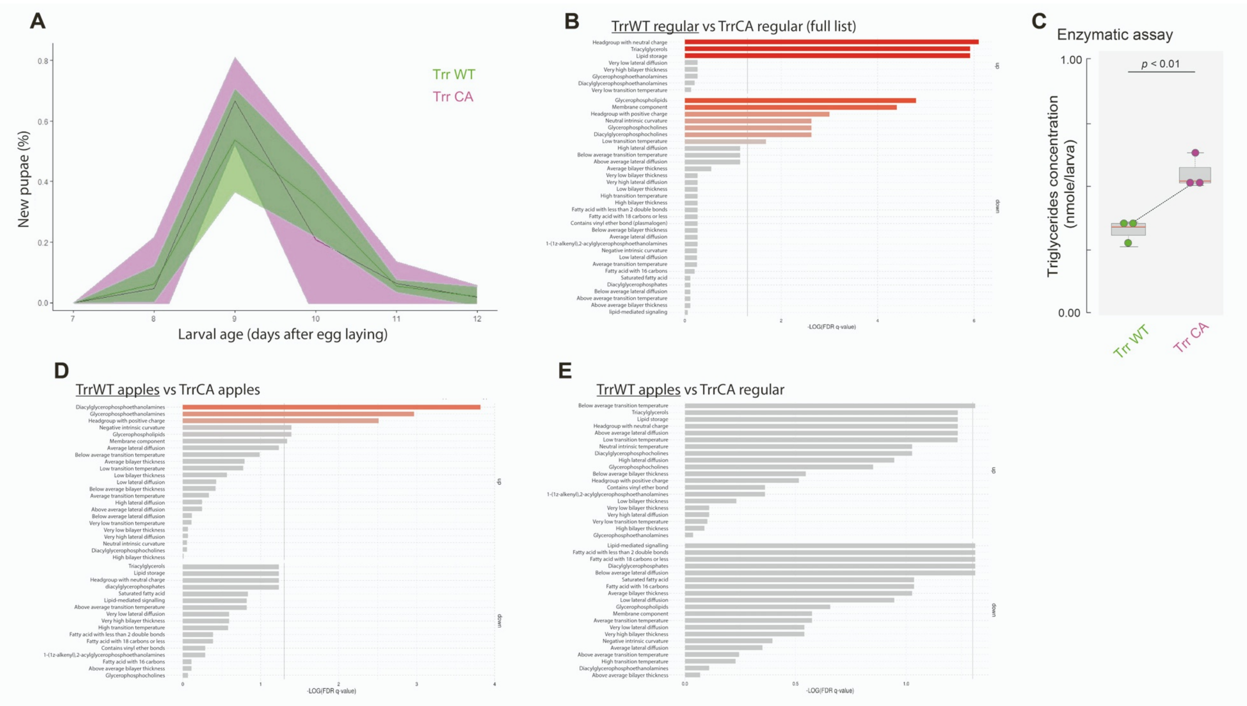

Supplementary Figure 5: Metabolomics of the *trr*[1] lines. Related to Figure 3.

(A) Pupariation time in both *trr*[1] mutant lines. The middle line shows the mean value for that time point, whilst the ribbon describes the CI (α=0.05); N (number of tested populations per condition) = 3.

(B) Enrichment analysis comparing both *trr*[1] mutant lines, based on the abundance values for 77 metabolites consistently detected in all tested conditions (full list)

(C) Enzymatic determination of triglyceride levels in TrrWT and TrrCA larvae with the same time of development (120 h). N=3. Centre line, mean; upper and lower limits, s.d.; whiskers, 2.s.d. 95% CIs. Two-tailed Student's *t*-test comparing the two *trr*[1] lines.

(D & E) Enrichment analysis from pairwise comparisons between *trr*^{1} mutant lines on apple-based food (D) and between the two populations with strong overlap in the PCA, TrrWT on apples and TrrCA on standard food (E).

gRNA1	GTCAAAGTTACAAGAAAACATGG
gRNA2	GCTATATGCAGATATGCTCGTGG
Template	TCAGAGCTAAGCAACCTGCTCCGTGTGGGCAACATGACGTTCCTGAACGTGGGTCAGTTACTGCCGCATCAGC<br>TGGAGGCCTTCCACACACCCCACTACATCTACCCCATTGGCTACAAGGTGGTAAGTACAAGTACCAGCCGGTC<br>GGCCGCCATGTTTATGAACAGCAATCTTAAATCCCGTTCCTCTTCCTCCCCCATAGAGTCGCTACTACTGGTGCG<br>TGCGACGACCCAATCGTAGGTGTCGATACATTTGCAGTATCGCAGAGGCCGGATGCAAGCCCGAGTTTCGCAT<br>CCAGGTGCAGGACGCCGGCGACAAGGAGCCGGAGCGCGAGTTTCGGGGCAGTTCGCCCTCGGCGGTCTGGC<br>AGCAGATTCTGCAGCCAATCACGCGACTGCGTAAGGTGCACAAGTGGTTGCAGCTCTTTCCCCAGCACATCAG<br>TGGTGAGGACCTATTTGGCCTAACGGAACCGGCCATTGTGCGAATCCTGGAAAGCCTCCCGGGCATTGAGAC<br>GCTCACTGACTACCGCTTCAAGTACGGTCGCAACCCGCTTCTTGAGTTCCCGCTAGCCATCAATCCGTCTGGAG<br>CGGCGCGTACGGAACCCAAGCAACGACAGCTGCTCGTCTGGAGGAAGCCGCATACGCAACGCACGGCCGGCA<br>GCTGCAGCACGCAGCGGATGGCCAACTCGGCTGCGATTGCCGGAGAAGTGGCCTGCCCGTACAGCAAGCAGT<br>TCGTGCATTCGAAGAGCTCCCAGTACAAAAAGATGAAGCAGGAGTGGCGCAACAACGTCTACTTGGCGAGGT<br>GAGACATTAGGTCCTAGTCAATAACCACTGATTACTTAAGCTCTCATTTTCTAGATCTAAGATCCAGGGTCTTG<br>GCTTGTATGCAGCTCGGGACATTGAAAAGCACACCATGATAATCGAGTACATTGGCGAGGTTATCCGCACCGA<br>GGTCTCCGAGATACGCGAGAAGCAGTACGAGTCCAAGGTGGGTTTCGCGACCATGCTTCCTTGTAACTTTGAC<br>TAATCTCTGATTCCCCAGAACCGTGGCATCTACATGTTCCGGCTGGATGAAGACCGCGTGGTGGACGCCACTCT<br>AAGCGGCGGGTTGGCGCGCTACATCAACCATTCGTGCAATCCCAACTGTGTTACCGAGATAGTGGAGGTCGAT<br>CGCGACGTGCGGATCATAATATTCGCCAAGCGGAAGATCTATCGCGGCGAGGAGGTGGGTCCCGCAAATAGA<br>AGTGTTTCGCCTGTACCTTAACTCACAATATCTCTTAAATTCTGTGCTTTAGCTCTCGTACGACTACAAGTTCGAC<br>ATTGAGGACGAGTCGCACAAGATACCG**GCC**GCCTGTGGAGCGCCCAACTGTCGCAAGTGGATGAACTAGAGT<br>CGGGAGGAGCCTCTAGCGGAGGACCAAAGGAAGCGCAAGAAGCGGCGGCGAGCACGTCGCTCCCAGTGAAC<br>AGGAACAGGAGTAGAGACAGGAACAGGAACAGGACTTGTATCTGATCAAGTAGCTAGATTCGCTAAGCATTA<br>TAGTTCGTGTACATTTATATATATATATATATATATATATATATATTTATATATATATAGTTTATATGCTATATGC<br>AGATACGCCCGTGGAGAGCATATGACCTTGTAAGTTTTGTATATGTGCCCACTGTAGCCAATTCGTATCCTTTA<br>TATCGCTCTTAAGTGCAAAAGTGGAGAGTGTCTTCATAGTTTAAATTGAATAATTGCTATCGTTTCTACATAGA<br>AAGAATATAAATGTTACTGCTAGGACACGACCACCCCTGTAATGTATTTCGGACCCCCTCTCCTCACACAGTCG<br>CTGGAGCACCTAATTTTATTTGTTTTAGCACCATCTCCACATTCATCACTACCACCATTGCAATTATCGCGAGGA<br>AACTGACCGCCCCGATGGCCAACCCAAAACTTGACTTTATGCGTGTGTGCCTTCCCCTCTGTTTACCATTATTAT<br>TATTATCATAATTGTAGCGGTTATCTTTCAATATTCGGCCACTTTACATGGGGAGCGATCCTTCTTTCGCTGCGT<br>GTATCGTGCGCAACCTTCTTTGAATATATACACTGATAAGGATATGAACACCTACTTCTATTTTATTTTATTTAAA<br>TAGTTTGCGATTACTTTTAAGTACATTAACTAACTCCGAACAACGTGTATTTTTCATCCCCTTAAATAGTAGCTA<br>AGTAAATTTTATGCCACCATTTTACGCACGTGATCTTTTATTCCAACTTATTGCAAACGGCAAACTGGAAATTAC<br>GAAATGGGGACGACAGATATTGCACCTAAGATCTTCGCTTGGCCAGCGCCAGAAGCCTCTGGAGCTGGACTG<br>CGGCAGCCAGGGTGAGTATGTCGAAGTTCTTCAGGTACCAGGAGATGTTGTACATGATGTGGATGCGCCAGT<br>TCTTGTCGAGGAACGCTTTGTAGAAGTGGCTCATCTGGTCCGCGGACACTTCGCTGGTTCCGCTTTCCTTGTGC<br>AGGCGGATGAAGTCTTCCTTTTCCTGTAAACGAACCAATTCGAAAGAGACGACATTAGCGGACTAGAAAGAGA<br>TGGCACAGTGGTCGTGGTTGTACCTCGTAGAAGCGCTTATTGTGTTTTGT

Supplementary table 1. Sequences employed to build a new TrrCA allele using CRISPR/Cas9. Related to Figure 4.

Sequences for the two guides and the template DNA that were required to edit the native *trr* locus using CRISPR/Cas9. The nucleotides in red are the ones that were altered to turn the Trr protein catalytically deficient by replacing the cysteine at position 2398 by an alanine. Guide-related sequences in the template are shown in green.
